# Atypical Ebola Virus Disease in a Nonhuman Primate following Monoclonal Antibody Treatment Is Associated with Glycoprotein Mutations within the Fusion Loop

**DOI:** 10.1128/mBio.01438-20

**Published:** 2021-01-12

**Authors:** Logan Banadyga, Wenjun Zhu, Shweta Kailasan, Katie A. Howell, Krzysztof Franaszek, Shihua He, Vinayakumar Siragam, Keding Cheng, Feihu Yan, Estella Moffat, Wenguang Cao, Anders Leung, Carissa Embury-Hyatt, M. Javad Aman, Xiangguo Qiu

**Affiliations:** a Special Pathogens Program, National Microbiology Laboratory, Public Health Agency of Canada, Winnipeg, Canada; b Integrated BioTherapeutics, Inc., Rockville, Maryland, USA; c Science and Technology Core, National Microbiology Laboratory, Public Health Agency of Canada, Winnipeg, Canada; d Key Laboratory of Jilin Province for Zoonosis Prevention and Control, Changchun Veterinary Research Institute, Chinese Academy of Agricultural Sciences, Changchun, Jilin, China; e Canadian Food Inspection Agency, National Centre for Foreign and Animal Disease, Winnipeg, Canada; La Jolla Institute for Immunology and The Scripps Research Institute; Washington University School of Medicine

**Keywords:** Ebola virus, Ebola virus disease, filovirus, glycoprotein, monoclonal antibody, pathogenesis, recrudescence

## Abstract

Ebola virus (EBOV) is responsible for numerous devastating outbreaks throughout Africa, including the 2013–2016 West African outbreak as well as the two recent outbreaks in the Democratic Republic of the Congo (DRC), one of which is ongoing. Although EBOV disease (EVD) has typically been considered a highly lethal acute infection, increasing evidence suggests that the virus can persist in certain immune-privileged sites and occasionally lead to EVD recrudescence. Little is understood about the processes that contribute to EBOV persistence and recrudescence, in part because of the rarity of these phenomena but also because of the absence of an animal model that recapitulates them. Here, we describe a case of EBOV persistence associated with atypical EVD in a nonhuman primate (NHP) following inoculation with EBOV and treatment with an experimental monoclonal antibody cocktail. Although this animal exhibited only mild signs of acute EVD, it developed severe disease 2 weeks later and succumbed shortly thereafter. Viremia was undetectable at the time of death, despite abundant levels of viral RNA in most tissues, each of which appeared to harbor a distinct viral quasispecies. Remarkably, sequence analysis identified a single mutation in glycoprotein (GP) that not only resisted antibody-mediated neutralization but also increased viral growth kinetics and virulence. Overall, this report represents the most thoroughly characterized case of atypical EVD in an NHP described thus far, and it provides valuable insight into factors that may contribute to EBOV persistence and recrudescent disease.

## Abstract (precis)

Ebola virus remains a global threat to public health and biosecurity, yet we still know relatively little about its pathogenesis and the complications that arise following recovery. With nearly 20,000 survivors from the 2013–2016 West African outbreak, as well as over 1,000 survivors of the recent outbreak in the DRC, we must consider the consequences of virus persistence and recrudescent disease, even if they are rare.

## INTRODUCTION

Ebola virus (EBOV), a member of the *Filoviridae* family, is the causative agent of a severe and often lethal syndrome known as Ebola virus disease (EVD) (1–4). In humans, EVD typically manifests after a 7- to 10-day incubation period with the sudden onset of nonspecific symptoms such as fever, malaise, and fatigue. As the infection progresses, virus spreads systemically, eventually infecting numerous organs and organ systems, including the liver, spleen, and kidney. Rampant viral replication leads to the destruction of these tissues and precipitates many of the gastrointestinal, respiratory, and vascular symptoms associated with the disease. Central nervous system dysfunction, including meningoencephalitis, is also common and may be a result of virus replication in these tissues. An uncontrolled and ineffective proinflammatory immune response does little to curtail viral replication and can further exacerbate tissue damage. Indeed, high peak viremia levels and a dysregulated cytokine response are both predictive of a poor outcome with EVD. Within 6 to 16 days after symptoms first appear, death usually occurs due to multiorgan failure and hypovolemic shock, whereas nonfatal cases begin to improve after 7 to 11 days.

EVD has historically been thought of as an acute disease, in the sense that patients either succumb soon after infection or face a protracted convalescence that results in long-lasting immunity to reinfection. In the wake of the massive 2013–2016 West African epidemic, however, it has since become apparent that EBOV can persist for long periods of time in certain body fluids of immune-privileged compartments, including the eyes, genital tract, and central nervous system (5–12). In particular, viral persistence in the semen of male survivors is well documented (4, 13, 14) and has been linked to sexual transmission of EBOV, sometimes months after initial recovery from disease (6, 15–18). In rare cases, EBOV persistence in an immune-privileged site has been associated with the reemergence of an organ-specific inflammatory disease in patients who have recovered from acute illness, a phenomenon sometimes referred to as recrudescence and exemplified in a few case reports. For instance, 9 months after recovering from severe EVD, a 39-year-old female patient experienced a relapse characterized by a severe, rapid-onset febrile disease and meningoencephalitis (11). Notably, while viral RNA levels in her blood remained very low, she exhibited higher levels in cerebrospinal fluid (CSF) samples, from which infectious virus was also isolated. A second case of organ-specific recrudescence was described in a 43-year-old male, who developed severe uveitis 14 weeks after EVD onset and 9 weeks after clearance of viremia (12). Infectious EBOV was detected in the aqueous humor of the patient’s eye, along with high levels of EBOV RNA. Based on these rare occurrences, it has been proposed that recrudescent EVD may be the consequence of very high levels of virus in blood “seeding” immune-privileged sites, such as the central nervous system (CNS) and eyes, from which secondary disease manifests (19). Importantly, like sexual transmission linked to persistent virus in semen, an EVD outbreak cluster in the Democratic Republic of the Congo has been linked to transmission from a suspected case of relapsed EVD (20, 21).

Given the severity of the disease and its propensity to cause large outbreaks, EBOV continues to pose a serious global public health threat—a threat proven by the West African epidemic and underscored by several additional outbreaks since then. Indeed, the recent outbreak in the North Kivu and Ituri provinces of the Democratic Republic of the Congo was the second largest in history, resulting in 3,470 cases and 2,280 deaths (22). Moreover, since June 2020, an ongoing outbreak centered on Mbandaka, also in the Democratic Republic of the Congo, has so far resulted in 110 cases and 47 deaths as of 1 September 2020 (23). The need for effective therapeutics to treat EVD therefore remains as pressing as ever, and many promising candidates have recently been developed. Among the most promising are monoclonal antibodies (mAbs) that target and neutralize the EBOV glycoprotein (GP), the sole surface-exposed viral protein that is responsible for virus attachment and entry into host cells. A variety of mAbs and mAb cocktails have been developed and initially evaluated in rodent and nonhuman primate (NHP) models (24), with Mab114 (a single antibody) (25) and REGN-EB3 (a cocktail of three antibodies) (26) shown to have superior efficacy in humans in a recent clinical trial conducted during the North Kivu/Ituri outbreak (27). Intriguingly, some cases of EVD recrudescence, such as those described above, have been associated with the use of EBOV-specific countermeasures, including mAb therapeutics and convalescent plasma (28). However, whether antibody-based therapeutics are directly linked to the development of EBOV persistence, recrudescence, or other clinical sequelae is a critical, but so far unanswered, question.

We recently described a pan-filovirus cocktail consisting of three neutralizing mAbs—CA45, FVM04, and MR191—that provided 100% protection to rhesus macaques infected with EBOV or the related filoviruses, Sudan and Marburg virus (29). During a parallel experiment, we observed a single NHP that experienced mild clinical signs of acute EVD after treatment with CA45 and FVM04 only to develop severe disease approximately 2 weeks later and succumb shortly thereafter. Remarkably, at the time of death, no virus could be detected in blood, but abundant levels of viral RNA were detected in most tissues, each of which appeared to contain distinct viral quasispecies. Sequencing analysis identified a single mutation, E545D in GP that not only resisted neutralization by CA45 but also increased viral growth kinetics and virulence. Together, these data provide insight into the development of atypical EVD and the factors that may contribute to EBOV persistence and recrudescence.

## RESULTS

### Atypical Ebola virus disease in a nonhuman primate.

**FIG 1:**
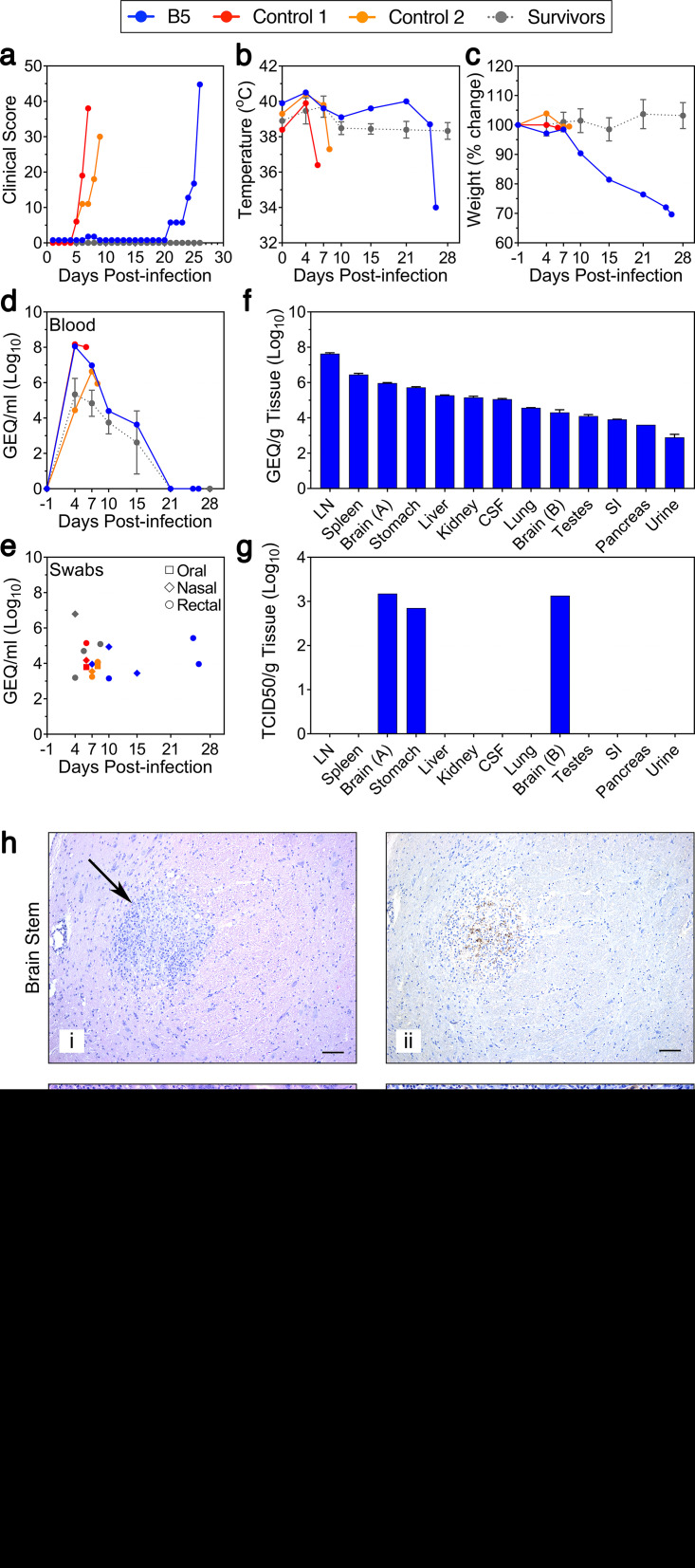
Atypical Ebola virus disease in a nonhuman primate. (a to c) Clinical health scores, rectal temperatures, and percent weight changes for NHP B5, Control 1, and Control 2. Clinical scores for the survivors are presented as mean values, and temperature and weight change are presented as mean values ± standard deviations (SD) (error bars). (d to f) Viral RNA loads (genome equivalents [GEQ] per milliliter or gram) in blood, swabs (oral, nasal, and rectal swabs), and various tissues. For swab samples, symbol shapes indicate type of sample and colors indicate NHP, consistent with other panels (i.e., blue, NHP B5; red, Control 1; orange, Control 2; gray, survivors). (g) Infectious virus loads (median tissue culture infectious dose per gram) in various tissues. LN, mesenteric lymph node; SI, small intestine. (h) Histopathology (i, iii, and v) and immunohistochemistry (ii, iv, and vi) in brain stem, stomach, and mesenteric lymph node samples. A single focus of inflammation was observed (arrow) in the brain stem (i), composed primarily of cells with histiocytic morphology. This lesion was associated with viral antigen (ii). Lesions in the stomach were composed of multiple cell types, including neutrophils, histiocytes, and multinucleated giant cells in both the submucosa (asterisk) and lamina propria (arrow) (iii); viral antigen was observed primarily in histiocytic cells (arrow) and in a few multinucleated cells (arrowhead) (iv). Central accumulations of neutrophils surrounded by a rim of histiocytes (arrowhead) and scattered multinucleated giant cells (arrows) were often observed within the mesenteric lymph node lesions (asterisk) (v), and viral antigen could be observed within all cell types within the lesion (vi). Bars, 100 μm (i and ii) and 50 μm (iii to vi).

To assess the efficacy of pan-ebolavirus and pan-filovirus antibody cocktails, rhesus macaques were inoculated with a lethal dose of EBOV and treated with different combinations and dosages of three mAbs: FVM04, CA45, and MR191 (see Table S1 in the supplemental material). Two untreated control animals developed severe EVD by 5 days postinfection (DPI) and were euthanized on 7 and 8 DPI, whereas all except one of the treated animals survived (Fig. 1a). A single macaque (NHP B5) treated with FVM04 and CA45 on 4 and 7 DPI exhibited mild signs of acute EVD (based on clinical scoring) but went on to develop a severe, late-onset disease that progressed rapidly and culminated in euthanasia at 26 DPI (Fig. 1; Table S2). Unlike all other treated animals, which remained clinically normal throughout the study, NHP B5 exhibited mild signs of disease on 7 and 8 DPI (Fig. 1a), preceded by a slight increase in body temperature on day 4 (Fig. 1b). From 9 DPI onwards, the animal returned to a baseline clinical score (Fig. 1a) despite diarrhea/soft stool from 8 to 12 DPI and weight loss, first noted on 10 DPI (Fig. 1c; Table S2). On 21 DPI, the animal’s condition began to rapidly deteriorate (Fig. 1a; Table S2), with the animal exhibiting decreased activity, depression, and anorexia—all of which worsened over the following 5 days. Given the animal’s history of diarrhea and weight loss, enrofloxacin (5 mg/kg of body weight) was administered on 24 and 25 DPI to treat a presumptive bacterial infection; however, subsequent reverse transcription-quantitative PCR (RT-qPCR) analyses targeting bacterial 16S rRNA were negative for all blood samples at all time points (data not shown). By 26 DPI, NHP B5 was found not moving and unresponsive, with a decreased respiration rate and hypothermia (Fig. 1a and b; Table S2). Accordingly, the animal was euthanized for reaching the humane endpoint clinical score.

10.1128/mBio.01438-20.8 TABLE S1 Study outline. Outline of experiments involving all animals analyzed in this study, including NHP B5, as well as the surviving animals and control animals. Group designation, along with study ID and animal ID are indicated. Age, sex, and weight at the start of the study are provided, as well as the EBOV inoculation dose and experimental treatment regimen. Outcome is indicated in the final column. Download Table S1, DOCX file, 0.01 MB. © Crown copyright 2021. 2021 Crown This content is distributed under the terms of the Creative Commons Attribution 4.0 International license.

10.1128/mBio.01438-20.9 TABLE S2 Clinical history of NHP B5. The clinical features of disease observed in NHP B5 throughout the experiment are detailed, according to day postinfection (DPI). Download Table S2, DOCX file, 0.01 MB. © Crown copyright 2021. 2021 Crown This content is distributed under the terms of the Creative Commons Attribution 4.0 International license.

Clinical signs of disease in NHP B5 were preceded on 4 DPI (prior to the first mAb treatment) by high levels of RNA in blood (7.8 log_10_ genome equivalents [GEQ]/ml), exceeding most of the other infected animals by more than 2 log units (Fig. 1d). Following treatment with FVM04 and CA45 on 4 and 7 DPI (Table S2), viral RNA levels in blood dropped substantially, demonstrating not only that the mAb cocktail was highly effective against EBOV but also that NHP B5 was not refractory to the treatment itself. Similar to the surviving animals, by 21 DPI, viral RNA was no longer detectable by RT-qPCR in blood of NHP B5 (Fig. 1d), and it remained undetectable even at the terminal stage of disease when the animal was sickest (Fig. 1a and d). Low levels of viral RNA were also detected sporadically in the oral, nasal, and rectal mucosa throughout the study, suggesting virus shedding, especially from the nose early during infection and the rectum later during infection (Fig. 1e). Notably, viral RNA was detected only in the nasal and rectal mucosa from the surviving NHPs up to 7 DPI, suggesting clearance of the virus and cessation of virus shedding in these animals (Fig. 1e).

Despite the absence of detectable viral RNA in blood of NHP B5 at the time of euthanasia, viral RNA was detected via RT-qPCR in a variety of tissue samples collected postmortem (Fig. 1f). The liver, spleen, kidney, and stomach all had relatively high levels of viral RNA, with the highest levels detected in the mesenteric lymph nodes. Notably, viral RNA was detected in two separate samples taken from the brain stem, as well as the cerebral spinal fluid and the testes, suggesting infiltration of EBOV into immune-privileged sites. The systemic presence of viral RNA was corroborated by *in situ* hybridization, which detected significant signal in the brain (meninges), stomach, mesenteric lymph node, and spleen (see Fig. S1a in the supplemental material). Infectious virus (as assessed by the presence of cytopathic effect) was detected in a minority of samples following titration of sample homogenates on Vero E6 cells (Fig. 1g). While the two brain samples exhibited infectious titers of 3.18 and 3.13 log_10_ 50% tissue culture infective dose (TCID_50_)/g and the stomach sample exhibited a titer of 2.85 log_10_ TCID_50_/g, none of the other samples produced infectious virus (Fig. 1g). The observed discrepancy resulting from the high levels of viral RNA but the inability to detect infectious virus may have resulted from different tissue sections containing different viral loads. It is also possible that some tissues retained an abundance of EBOV genetic material left over from an already cleared infection or that these tissues were the productive site of defective interfering genomes rather than infectious virus.

10.1128/mBio.01438-20.1 FIG S1 *In situ* hybridization and additional histopathology in NHP B5. (a) *In situ* hybridization revealed positive staining in the meninges (i) which often corresponded to large cells with histiocytic morphology (ii, arrow). Positive staining in the stomach (iii) was associated with a lesion (iv) in both the submucosa (arrow) and lamina propria (arrowhead). In the mesenteric lymph node, RNA was detected within lesions (v) that were primarily associated with large cells with histolytic morphology (vi). In the spleen, positive staining was limited to small areas within the white pulp germinal centers (vii, arrows) and was often observed adjacent to the central artery (viii). Scale bars represent 100 μm (i), 20 μm (ii and viii), 200 μm (iii and vii), 50 μm (iv and vi), and 500 μm (v). (b) Histopathology (i, iii, v, and vii to ix) and immunohistochemistry (ii, iv, and vi) in brain stem, stomach, mesenteric lymph node, small intestine, and lung samples. Nonsuppurative meningitis was observed (i, arrows), and viral antigen was detected in these areas (ii, arrows). In the sections of stomach examined, a single focus of inflammation was observed (iii) in the submucosa (asterisks) and extending into the lamina propria (arrow). In the mesenteric lymph node, there were multifocal areas of pyogranulomatous inflammation (v, arrows) that were associated with the presence of viral antigen (vi). In the small intestine, the lamina propria exhibited increased numbers of inflammatory cells, including lymphocytes, plasma cells, and neutrophils (vii). There are numerous necrotic cells with shrunken cytoplasm and pyknotic nuclei (vii, arrow), and siderophages (vii, arrowheads) are scattered throughout, indicating previous hemorrhage. In the lung, multifocal areas of pulmonary hemorrhage and congestion were observed (viii). At a higher magnification, evidence of interstitial pneumonia was present, characterized by alveolar septa that are hypercellular and expanded by infiltration of inflammatory cells, including neutrophils and macrophages (ix, arrow). Areas of edema (ix, asterisk) and hemorrhage were also observed in the lung. Virus antigen was not detected by immunohistochemistry in the small intestine and lung samples. Scale bars represent 20 μm (vii), 50 μm (ix), 100 μm (i and ii), 200 μm (iii and iv), and 500 μm (v, vi, and viii). Download FIG S1, TIF file, 2.9 MB. © Crown copyright 2021. 2021 Crown This content is distributed under the terms of the Creative Commons Attribution 4.0 International license.

Immunohistochemistry analysis corroborated the systemic spread of virus, demonstrating the presence of virus antigen in the brain stem and stomach that was associated with inflammatory lesions (Fig. 1h; Fig. S1b). Interestingly, viral antigen was also detected in the mesenteric lymph node associated with pyogranulomatous inflammatory lesions, even though infectious virus could not be detected from similar samples (Fig. 1h; Fig. S1b). Virus antigen was not detected in tissue samples from the liver, kidney, small intestine, lung, or eye (data not shown).

Histopathology analysis revealed significant lesions in a number of tissues, some of which were not typical of EVD. Lesions observed in the brain stem were indicative of nonsuppurative meningoencephalitis (Fig. 1h; Fig. S1b), consistent with the type of inflammation observed with viral infections. Depletion of lymphocytes in the gut-associated lymphoid tissue of the stomach and mesenteric lymph node were observed and are consistent with EBOV infection; however, the pyogranulomatous lesions observed in the stomach and mesenteric lymph node were unusual (Fig. 1h; Fig. S1b). Although numerous macrophages containing abundant viral antigen were observed in these lesions, there were also large accumulations of neutrophils and scattered multinucleated giant cells, suggesting that the lesions were chronic in nature. The presence of bacteria was not detected by acid fast stain (Zeihl-Neelson) or modified Brown and Brenn Gram stain (data not shown). Within the lamina propria of the small intestine, there was an increased amount of inflammatory cells (primarily lymphocytes and plasma cells with some neutrophils) with multifocal areas of necrosis and the presence of abundant siderophages (Fig. S1b). Although the presence of siderophages suggests some chronicity, these cells can be present within 3 to 4 days. Again, no evidence of bacteria was detected in these samples, and these lesions are not typical of the common causes of diarrhea in NHPs. Acute lesions of hemorrhage and interstitial pneumonia were observed in the lung (Fig. S1b), and although no viral antigen was detected, there was no indication of any other etiology. Notably, no significant lesions were observed in the liver, kidney, or spleen samples obtained from NHP B5 (data not shown), suggesting the resolution of any damage that might have occurred within the first few days following infection.

**FIG 2:**
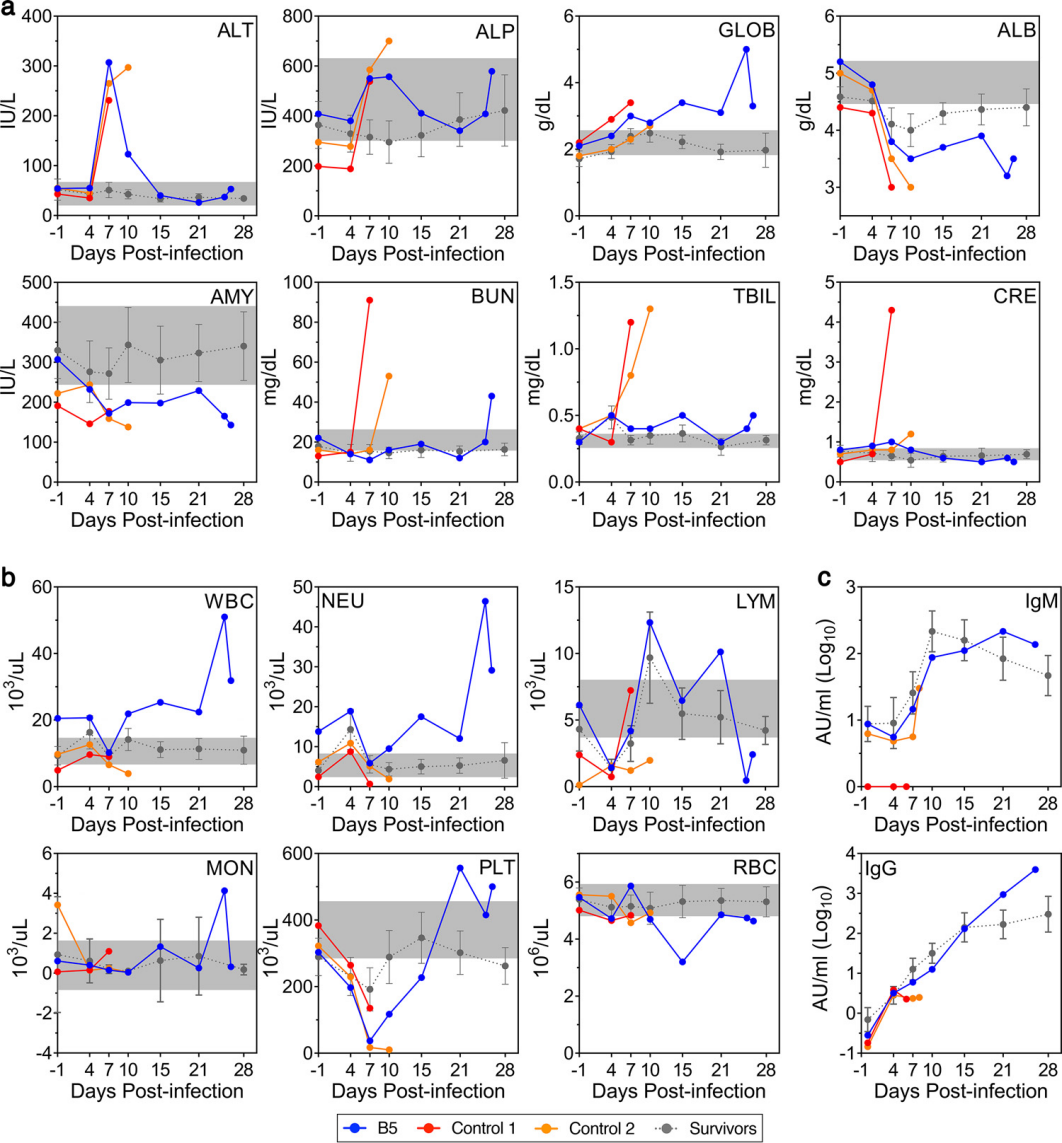
Clinical chemistry, hematology, and serology from NHP B5. Blood/plasma samples obtained from NHP B5, Control 1, Control 2, and all survivors at various time points throughout infection were analyzed for the indicated clinical chemistry (a), hematology (b), or serology (c) parameters. Data for the survivors (*n* = 14) are presented as mean values ± SD. Normal ranges for each clinical chemistry or hematology parameter are indicated in gray. ALT, alanine aminotransferase; IU/L, international units/liter; ALP, alkaline phosphatase; GLOB, globulin; ALB, albumin; AMY, amylase; BUN, blood urea nitrogen; TBIL, total bilirubin; CRE, creatinine; WBC, white blood cells; NEU, neutrophils; LYM, lymphocytes; MON, monocytes; PLT, platelets; RBC, red blood cells; IgM, immunoglobulin M; IgG, immunoglobulin G.

The clinical chemistry profile of NHP B5 was consistent with severe EVD in many respects, exhibiting several similarities with the control animals (Fig. 2a). A spike in alanine aminotransferase (ALT) levels at 7 DPI was particularly pronounced, although disturbances in alkaline phosphatase (ALP), albumin (ALB), and amylase (AMY) levels were also noted early postinfection. Globulin (GLOB) levels increased throughout the disease course, peaking on 25 DPI, while blood urea nitrogen (BUN) levels remained within the normal range throughout the study until spiking on 26 DPI, along with a second spike in ALP and another decrease in AMY and ALB. Notably, the later disturbances in clinical chemistry coincided with the animal’s rapidly deteriorating clinical condition (Fig. 1a). Curiously, total bilirubin (TBIL), creatinine (CRE), and calcium levels in NHP B5 did not appear to change dramatically, unlike in the control animals (Fig. 2a; Fig. S2). Fluctuations in additional analytes were also observed, both early and late postinfection, further suggestive of liver, kidney, and pancreas damage (Fig. S2). In contrast to NHP B5, the majority of clinical chemistry parameters in the survivors remained within the normal range throughout the study, reflecting the absence of disease in these animals (Fig. 2a; Fig. S2).

10.1128/mBio.01438-20.2 FIG S2 Additional clinical chemistry parameters from NHP B5. Blood/plasma samples obtained from NHP B5, Control 1, Control 2, and all survivors at various time points throughout infection were analyzed for the indicated clinical chemistry parameters. Data for the survivors are presented as mean values ± SD. Normal ranges for each clinical chemistry or hematology parameter are indicated in gray. GLU, glucose; TP, total protein; Na^+^, sodium; K^+^, potassium; Phos, inorganic phosphate; Ca^2+^, calcium. Download FIG S2, TIF file, 0.3 MB. © Crown copyright 2021. 2021 Crown This content is distributed under the terms of the Creative Commons Attribution 4.0 International license.

Hematological findings in NHP B5 were suggestive of immune dysfunction and typical of severe EVD (Fig. 2b). A drop in the white blood cell (WBC) and neutrophil counts on 7 DPI was followed by late-stage neutrophilic leukocytosis that coincided with clinical deterioration. Of note, NHP B5 baseline (day −1) WBC and neutrophil levels were moderately above the normal range (Fig. 2b). Lymphopenia was observed on 4 DPI, followed by a rebound in lymphocyte numbers and then a sharp decrease at the terminal stage of disease. Monocyte numbers remained within the normal range throughout the study, except for a spike at 25 DPI. Severe thrombocytopenia was observed between 4 and 15 DPI, after which platelet numbers increased and remained relatively high. Anemia appeared to develop after 7 DPI and became most severe on 15 DPI. In general, the control animals and survivors showed similar changes early during infection; however, the survivors eventually recovered and stabilized. Notably, NHP B5 did develop an EBOV GP-specific IgM and IgG response that mirrored or exceeded what was observed in the surviving animals (Fig. 2c), as measured by quantitative antibody binding.

**FIG 3:**
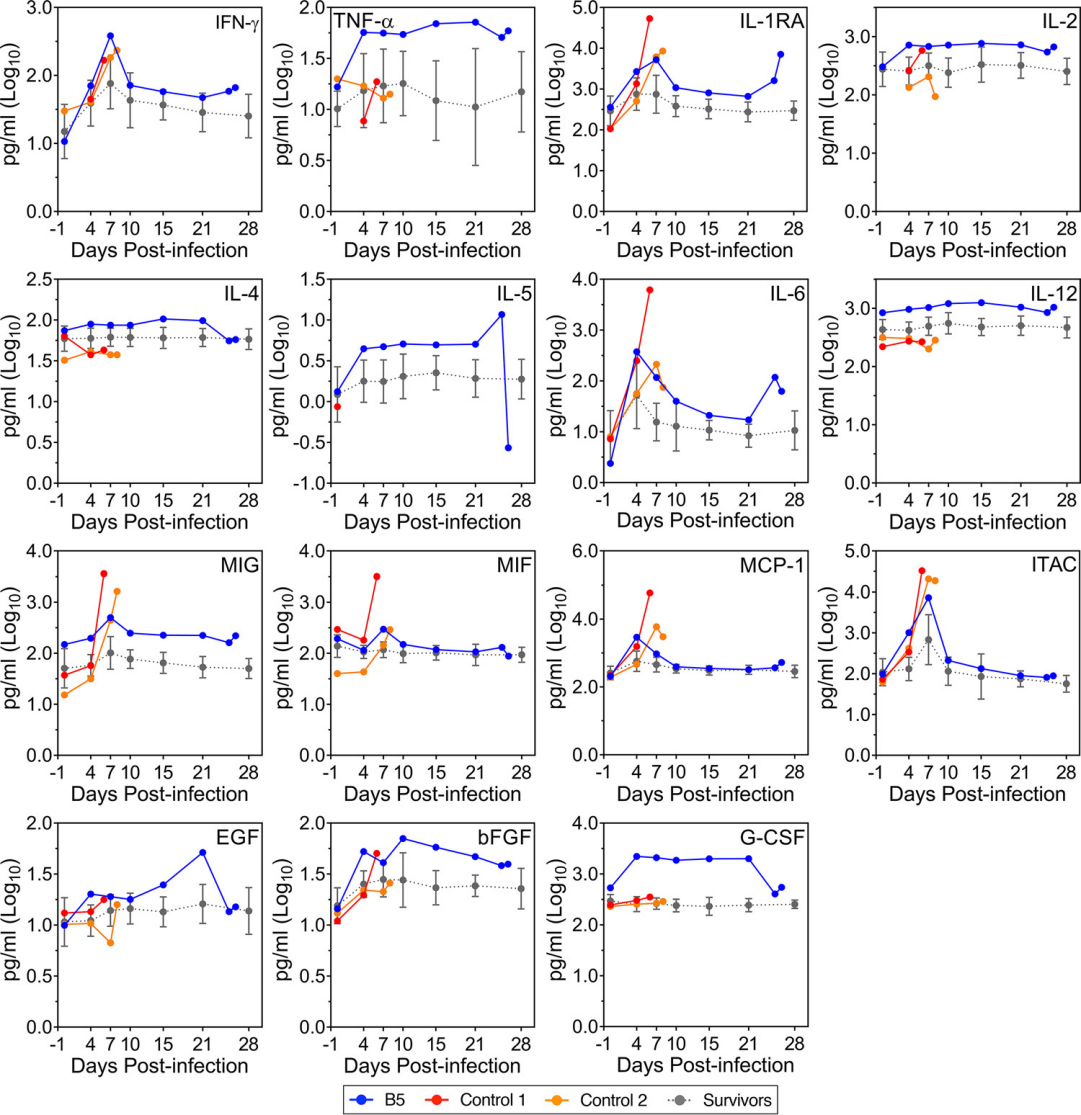
Cytokine profile disturbances in NHP B5. Plasma samples obtained from NHP B5, Control 1, Control 2, and all survivors at various time points throughout infection were analyzed for the indicated cytokine levels. Data for the survivors (*n* = 14) are presented as mean values ± SD. IFN-γ, gamma interferon; TNF-α, tumor necrosis factor alpha; IL-1RA, interleukin-1 receptor antagonist; IL, interleukin; MIG, monokine induced by IFN-γ; MIF, macrophage migration inhibitory factor; MCP-1, monocyte chemoattractant protein 1; ITAC, IFN-inducible T-cell alpha chemoattractant; EGF, epidermal growth factor; bFGF, basic fibroblast growth factor; G-CSF, granulocyte colony-stimulating factor.

Analysis of serum cytokine levels in NHP B5 revealed a pattern of significant immune dysregulation (Fig. 3). Gamma interferon (IFN-γ), interleukin-1 receptor antagonist (IL-1RA), interleukin-6 (IL-6), monokine induced by IFN-γ (MIG), macrophage migration inhibitory factor (MIF), monocyte chemoattractant protein 1 (MCP-1), and IFN-inducible T-cell alpha chemoattractant (ITAC) all exhibited discrete peaks early postinfection, resembling peaks observed in the control animals. Similarly, basic fibroblast growth factor (bFGF) increased immediately after infection and peaked on 10 DPI, before decreasing over the remainder of the infection. In contrast, IL-4, IL-5, IL-6, and epidermal growth factor (EGF) reached peak levels late during infection, between 15 and 25 DPI, before dropping considerably at the terminal time point. Tumor necrosis factor alpha (TNF-α), IL-2, and granulocyte colony-stimulating factor (G-CSF) increased markedly following infection and remained relatively high throughout, with the exception of G-CSF, which dropped dramatically on 25 DPI. On the other hand, levels of IL-12 remained relatively high throughout the study, even prior to infection. Less dramatic disturbances were also noted in levels of IFN-inducible protein 10 (IP-10), eotaxin, IL-8, and vascular endothelial growth factor (VEGF) in relation to the surviving animals, whereas several other cytokines, including IL-1β and macrophage inflammatory protein-1α (MIP-1α), showed relatively little change (Fig. S3).

10.1128/mBio.01438-20.3 FIG S3 Additional cytokine levels in NHP B5. Plasma samples obtained from NHP B5, Control 1, Control 2, and all survivors at various time points throughout infection were analyzed for the indicated cytokine levels. Data for the survivors are presented as mean values ± SD. IL, interleukin; MIP, macrophage inflammatory protein; IP-10, interferon-inducible protein 10; RANTES, regulated-on activation normal T-cell expressed and secreted; MDC, macrophage-derived chemokine; HGF, hepatocyte growth factor; VEGF, vascular endothelial growth factor; GM-CSF, granulocyte-macrophage colony-stimulating factor. Download FIG S3, TIF file, 0.6 MB. © Crown copyright 2021. 2021 Crown This content is distributed under the terms of the Creative Commons Attribution 4.0 International license.

### EBOV genome sequencing reveals distinct viral quasispecies.

**FIG 4:**
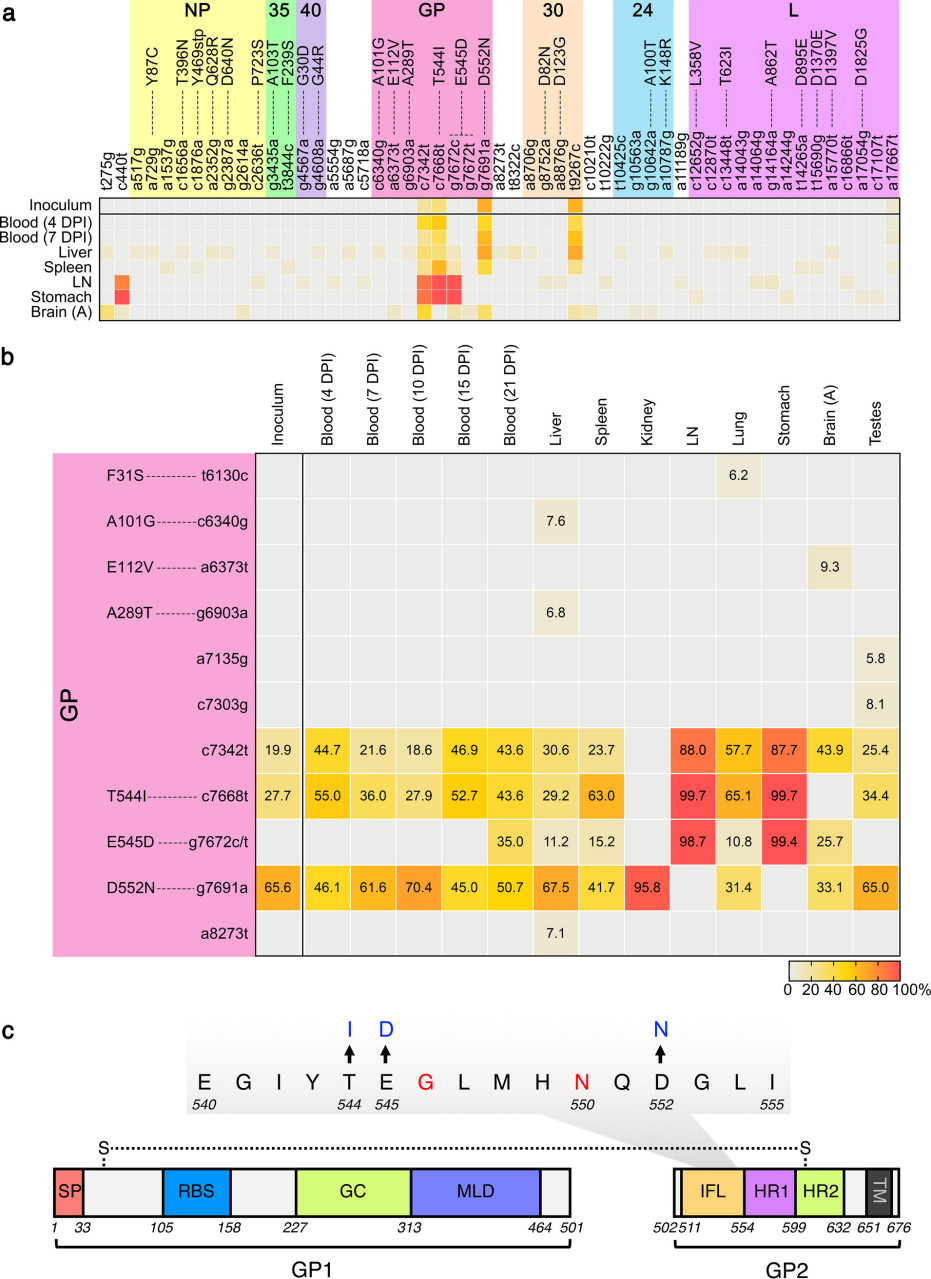
Genome mutations in virus from NHP B5. (a and b) Viral RNA isolated from the virus inoculum or the indicated blood or tissue samples was subjected to next-generation sequencing. All mutations that occurred at a frequency of 5% or higher across the entire genome (a) or within the GP coding sequence (b) are indicated by heatmap, with precise frequencies provided for the latter. Mutations are defined by the change in nucleotide at a given position on the genome using single-letter lowercase code and, where applicable, the change in amino acid at a given position within the specified protein using single-letter uppercase code. (c) A diagram of the GP1-GP2 heterodimer with amino acid sequence from the internal fusion loop highlighted. Mutations T544I, E545D, and D552N are indicated. G546 and N550 are highlighted in red and indicate critical amino acids within the CA45 epitope. DPI, days postinfection; LN, mesenteric lymph node; SP, signal peptide; RBS, receptor binding site; GC, glycan cap; MLD, mucin-like domain; IFL, internal fusion loop; HR, heptad repeats; TM, transmembrane region.

Deep sequencing of virus genomes obtained from several blood and tissue samples taken from NHP B5 identified numerous synonymous and nonsynonymous mutations, although the majority of these occurred at frequencies less than 10% (Fig. 4a; Fig. S4a). Only a few mutations were observed at frequencies higher than 10%, and most of these were located in GP (Fig. 4a; Fig. S4a). To obtain a more detailed understanding of the GP mutations within NHP B5, we deep sequenced the GP coding region only from a variety of additional samples, including the kidney, lung, and testes (Fig. 4b), which showed relatively high levels of viral RNA by RT-qPCR (Fig. 1f), as well as numerous blood samples (Fig. 4b). Notably, although blood was negative by RT-qPCR analysis on 21 DPI (Fig. 1d), we were able to amplify the GP open reading frame from this time point and include it in the sequence analysis. We suspect that the different PCR methodologies used for RT-qPCR and deep sequencing account for this discrepancy. Three nonsynonymous mutations in GP—T544I, E545D, and D552N—were found within the GP internal fusion loop (IFL), which comprises the binding epitope for antibody CA45 (Fig. 4c) (30); however, the frequency of each of these mutations differed widely depending on the tissue from which virus was sequenced (Fig. 4b). With the exception of the kidney and brain, T544I was represented to some degree in virus from all blood and tissue samples analyzed, and in particular, it predominated in virus from the mesenteric lymph node and stomach, where the frequency was greater than 99%. Sequencing of the inoculum virus revealed a small proportion of virus with T544I, indicating that this mutation existed prior to infection of NHP B5. D552N was found in a relatively high proportion of inoculum virus, and it was also found in virus from almost every sample analyzed, with the exception of the mesenteric lymph node and stomach. E545D was the only high-frequency mutation that was not detected in the inoculum virus, implying that it arose *de novo* during the course of infection. E545D was first detected in a blood sample from 21 DPI, despite the fact that no viral RNA was detected by RT-qPCR (Fig. 1d), suggesting very low levels of virus, perhaps seeded by replication in certain tissue compartments. Indeed, with the exception of the kidney and testes, E545D was detected in virus from almost all tissue samples taken at the terminal time point, including from the mesenteric lymph node and stomach, where the mutation frequency was close to 100%. Interestingly, the nucleotide mutation that resulted in c7342t was polymorphic, with the majority of sequence reads containing g7672c and a minority of reads (only from the brain and blood at 21 DPI) possessing g7672t (Fig. S4b). A single synonymous mutation at GP nucleotide position 7342 (c7342t; amino acid T435) was observed in a minority of inoculum virus and present in every tissue sample except the kidney at frequencies ranging from around 18% to nearly 90% in the mesenteric lymph node and stomach (Fig. 4b). While we did not subject the CSF sample to deep sequencing, we did identify two nonsynonymous GP mutations—T544I and E545D—along with the synonymous mutation c7342t by Sanger sequencing (data not shown), suggesting a profile similar to the sequences identified in the mesenteric lymph node and stomach.

10.1128/mBio.01438-20.4 FIG S4 Additional next-generation sequencing (NGS) data. Viral RNA isolated from the virus inoculum or the indicated blood or tissue samples was subjected to next-generation sequencing. (a) All mutations across the entire genome in samples from NHP B5 that occurred at a frequency of 2% or higher. (b) The nucleotide mutation at genome position 7672 was polymorphic, with both g-to-a and g-to-c mutations identified, both resulting in amino acid mutation E545D. The top two rows of the heatmap show nucleotide mutation frequency for each individual nucleotide mutation, while the bottom row shows the combined frequency. (c) All mutations across the entire genome in blood samples from control or surviving NHPs that occurred at a frequency of 5% or higher. (d) All mutations within the GP coding sequence in blood samples from control or surviving NHPs that occurred at a frequency of 5% or higher. Mutations are defined by the change in nucleotide at a given position on the genome using single-letter lowercase code. DPI, days postinfection; LN, mesenteric lymph node; T, terminal time point. Download FIG S4, TIF file, 1.2 MB. © Crown copyright 2021. 2021 Crown This content is distributed under the terms of the Creative Commons Attribution 4.0 International license.

Outside of the GP coding sequence, only three other mutations were detected at frequencies greater than 10%. A single synonymous mutation in VP30 (t9267c; amino acid S253) was present in the inoculum virus at a frequency of 64.8% and persisted to some degree in virus from most tissues sampled (Fig. 4a). A c440t mutation in the 5′ noncoding region of NP was detected in virus from the mesenteric lymph node and stomach, with frequencies of ∼82% and ∼99%, respectively (Fig. 4a). Virus from the brain also possessed the c440t mutation, albeit at a frequency less than 10%, as well as a nearby t275g mutation at a frequency of ∼30%. Notably, virus sequences obtained from blood of the control animals and survivors revealed a frequency of mutations that closely reflected the preexisting mutations identified in the inoculum virus, at least up to 7 DPI (Fig. S4c and d).

### Mutations in EBOV GP contribute to escape from antibody neutralization.

**FIG 5:**
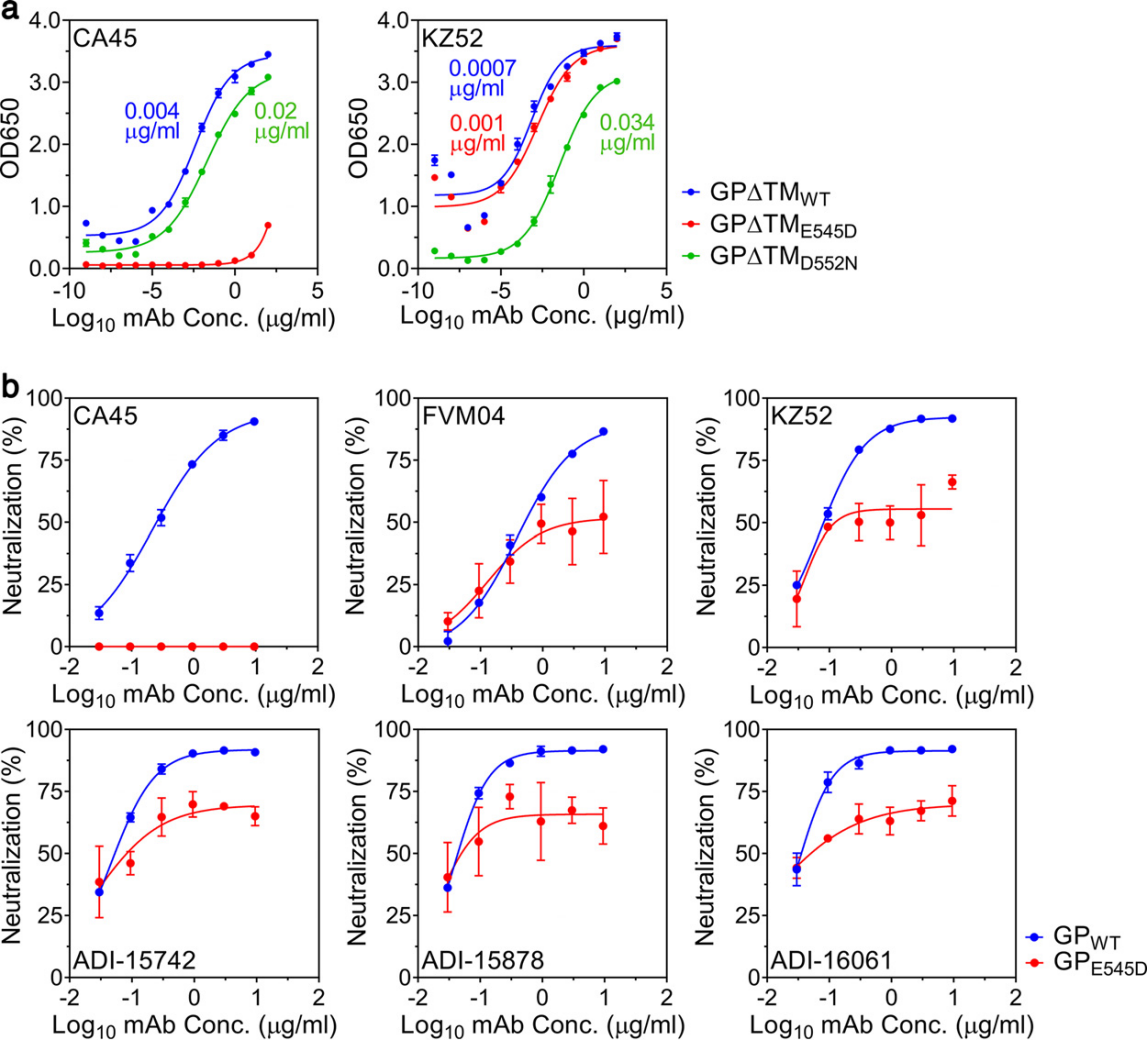
E545D mutation in GP prevents CA45 binding and neutralization. (a) Reactivity of CA45 and KZ52 to the wild-type (WT) EBOV GP ectodomain (GPΔTM_WT_) or GP ectodomains containing the E545D (GPΔTM_E545D_) or D552N (GPΔTM_D552N_) mutation was measured by ELISA. Median effective concentration (EC_50_) values are indicated for each curve. Data are presented as the mean optical density at 650 nm (OD650) ± SD from two replicates. (b) Replication-incompetent VSV expressing a luciferase reporter and pseudotyped with either WT EBOV GP (GP_WT_) or GP containing the E545D (GP_E545D_) mutation was incubated with the indicated antibodies at various concentrations before being used to infect Vero cells (60,000 cells/well) at a MOI of 0.04. Luciferase activity was measured 24 h later and used to calculate percent neutralization ± SD. Data are representative of three replicates.

FVM04 binds to the receptor binding site (RBS) on the apical side of GP (31), while CA45 recognizes a conformational epitope that consists of the IFL and the N terminus of GP1 (30, 32) (Fig. 4c). Since virus from NHP B5 exhibited a concentration of nonsynonymous mutations within the IFL and E545D was the only newly emerging mutation in this animal, we hypothesized that the E545D mutation may convey resistance to CA45 and allow viral escape. Indeed, CA45 bound strongly to the wild-type (WT) EBOV GP ectodomain (GPΔTM_WT_ [TM stands for transmembrane]), as assessed by enzyme-linked immunosorbent assay (ELISA), but it bound extremely poorly to the GP ectodomain harboring the E545D mutation (GPΔTM_E545D_) (Fig. 5a). Conversely, mAb KZ52 (33), used here as a control, showed no deficiency in binding to either GPΔTM_WT_ or GPΔTM_E545D_, suggesting that the E545D mutation was specific to CA45 (Fig. 5a). Binding of CA45 to a GP ectodomain containing the D552N mutation (GPΔTM_D552N_) was slightly reduced, while binding of KZ52 was moderately reduced (Fig. 5a). These results were corroborated by bio-layer interferometry, which revealed a >13-fold reduction in CA45 binding kinetics to GPΔTM_E545D_ (equilibrium dissociation constant [*K_D_*] = 32.5 ± 0.6 nM) compared to GPΔTM_WT_ (*K_D_* = 2.43 ± 0.3 nM) (Fig. S5). Binding of CA45 to GPΔTM_D552N_, on the other hand, was essentially unaffected (*K_D_* = 1.97 ± 0.4 nM) (Fig. S5). The neutralizing capacity of CA45 and FVM04—as well as several other potent neutralizing antibodies—was then evaluated against replication-incompetent vesicular stomatitis viruses (VSV) pseudotyped with EBOV wild-type GP (GP_WT_) or GP with the E545D mutation (GP_E545D_). Consistent with our hypothesis, the E545D mutation completely abrogated the neutralizing activity of CA45 (Fig. 5b). FVM04 (31), KZ52, ADI-15742 (34), ADI-15878 (34), and ADI-16061 (34) were all able to neutralize VSV-EBOV-GP_E545D_; however, these antibodies left a nonneutralized fraction ranging between 30 and 50% (Fig. 5b). Together, these data provide convincing evidence to suggest that the E545D mutation, but not the D552N mutation, arose as an escape mutant in response to the selective pressure from CA45 treatment.

10.1128/mBio.01438-20.5 FIG S5 E545D mutation reduces CA45 binding kinetics. (a) Bio-layer interferometry sensograms depicting the binding kinetics of CA45 with the wild-type (WT) EBOV GP ectodomain (GPΔTM_WT_) or GP ectodomains containing the E545D (GPΔTM_E545D_) or D552N (GPΔTM_D552N_) mutation. (b) Binding kinetics parameters for each GP construct, including on-rate (*k*_on_), off-rate (*k*_off_), and *K_D_*. Download FIG S5, TIF file, 0.5 MB. © Crown copyright 2021. 2021 Crown This content is distributed under the terms of the Creative Commons Attribution 4.0 International license.

### E545D mutation in EBOV GP contributes to virulence.

**FIG 6:**
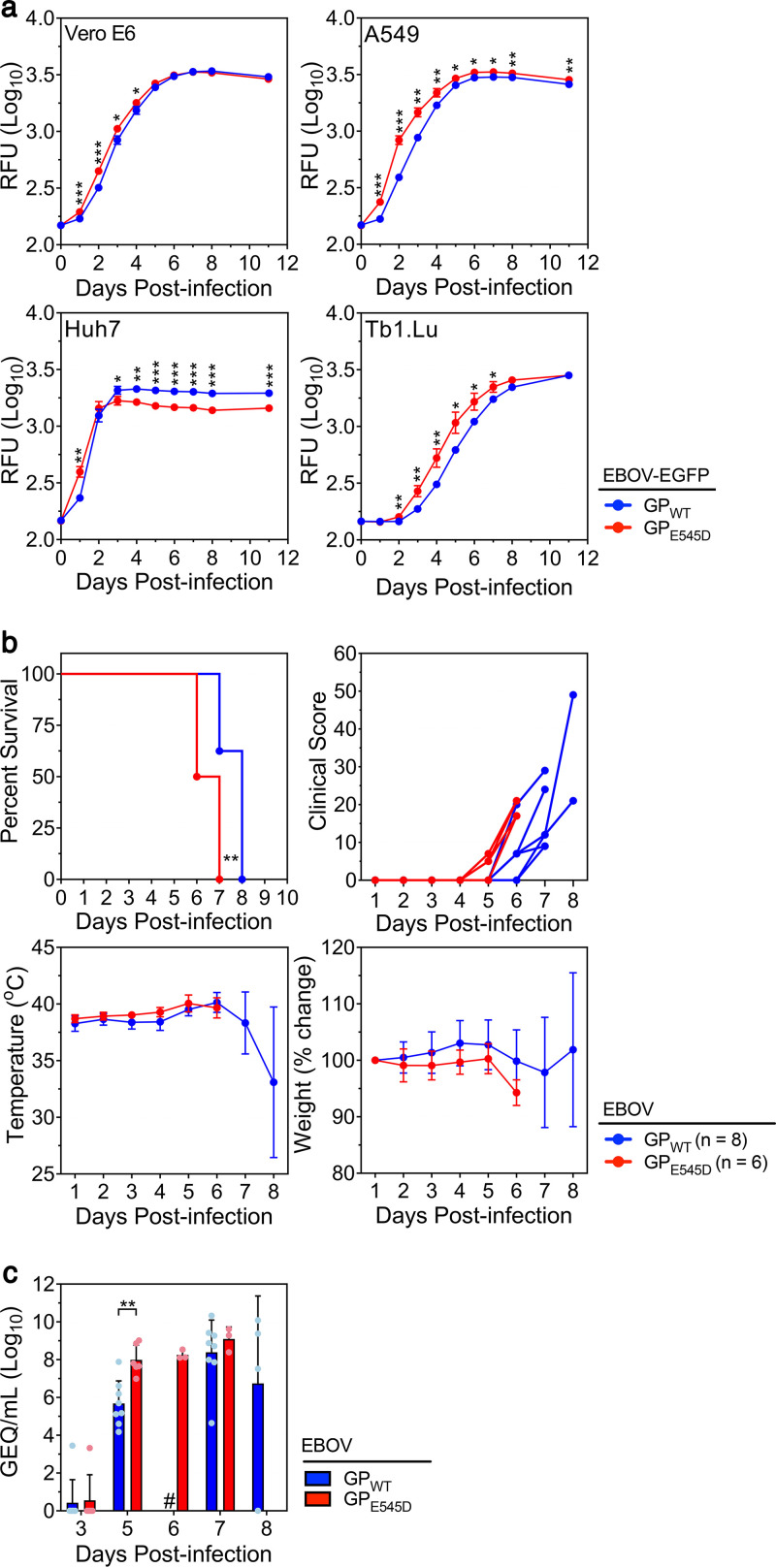
E545D mutation in GP enhances virus growth and contributes to virulence. (a) Vero E6, A549, Huh7, or Tb1.Lu cells were infected with recombinant EBOV expressing EGFP and wild-type (WT) GP (EBOV-EGFP-GP_WT_) or a GP containing the E545D mutation (EBOV-EGFP-GP_E545D_) at a MOI of 0.05 and EGFP fluorescence was measured at the indicated time points after infection. Data are presented as the mean relative fluorescence unit (RFU) ± SD from three technical replicates. *, *P* ≤ 0.05; **, *P* ≤ 0.01; ***, *P* ≤ 0.001; ****, *P* ≤ 0.0001. (b) Virulence was assessed in ferrets infected with a target dose of 1,000 TCID_50_ of either EBOV-GP_WT_ (*n* = 8) or EBOV-GP_E545D_ (*n* = 6). Percent survival, clinical score, body temperature, and percent weight change are presented. Clinical score data are presented for individual animals, while temperature and weight change are presented as mean values ± SD. Survival curves were significantly different (**, log rank, *P* = 0.0053; **, Gehan-Breslow-Wilcoxon, *P* = 0.0051). (c) Viral RNA levels in infected ferrets were assessed by RT-qPCR and are presented as genome equivalents (GEQ) per milliliter. Histograms indicate mean values ± SD, and dots indicate values from individual animals. **, *P* = 0.0015; #, animals not sampled.

Although the E545D mutation in GP clearly reduced CA45 neutralizing activity, we wondered whether this mutation might also affect viral growth kinetics. To this end, we generated a recombinant WT-EBOV expressing enhanced green fluorescent protein (EGFP) (WT-EBOV-EGFP) as well as a mutant EBOV expressing the GP E545D mutation in addition to EGFP (EBOV-EGFP-GP_E545D_). Vero E6, A549 (human lung epithelial), Huh7 (human liver epithelial), or Tb1.Lu (free-tailed bat lung epithelial) cells were infected with virus at a multiplicity of infection (MOI) of 0.05, and EGFP fluorescence was measured daily as an indication of virus growth (Fig. 6a). EBOV-EGFP-GP_E545D_ displayed significantly higher levels of EGFP fluorescence than WT-EBOV-EGFP in all cell lines tested early postinfection (∼1 to 3 DPI), suggesting that GP_E545D_ may confer a growth advantage to the virus in these cell lines. This difference disappeared in Vero E6 and Tb1.Lu cells at later time points (5 and 8 DPI, respectively) but persisted in A549 cells. In Huh7 cells, the growth advantage seemed to be lost by 3 DPI; however, we attribute this result to the fact that the mutant virus produced much greater cytopathic effect in Huh7 cells by 2 and 3 DPI and exhausted the cell monolayer sooner than WT-EBOV, a conclusion that is consistent with results obtained from a second experiment repeated at a lower MOI (Fig. S6).

10.1128/mBio.01438-20.6 FIG S6 E545D mutation in GP enhances virus growth. Vero E6, A549, Huh7, or Tb1.Lu cells were infected with recombinant EBOV expressing EGFP and wild-type (WT) GP (EBOV-EGFP-GP_WT_) or a GP containing the E545D mutation (EBOV-EGFP-GP_E545D_) at a MOI of 0.01, and EGFP fluorescence was measured at the indicated time points after infection. Data are presented as the mean relative fluorescence unit (RFU) ± SD. *, *P* ≤ 0.05; **, *P* ≤ 0.01; ***, *P* ≤ 0.001; ****, *P* ≤ 0.0001. Download FIG S6, TIF file, 0.2 MB. © Crown copyright 2021. 2021 Crown This content is distributed under the terms of the Creative Commons Attribution 4.0 International license.

To assess whether the increase in replication kinetics conferred by GP E545D contributed to a difference in virus virulence and/or fitness, we evaluated the mutant virus in ferrets, which are highly susceptible to WT-EBOV (35, 36). All animals infected with EBOV-GP_E545D_ exhibited clinical signs of disease 1 or 2 days before those infected with WT-EBOV and died approximately 1 day sooner, on 6 or 7 DPI (Fig. 6b). Although temperature, weight change, and hematological parameters did not vary considerably between the two groups (Fig. 6b; Fig. S7), certain clinical biochemistry parameters, namely, ALT, ALP, and GLOB, seemed to peak earlier and at higher levels in animals infected with EBOV-GP_E545D_ compared to those infected with WT-EBOV (Fig. S7a). Consistent with the EBOV *in vitro* growth kinetics experiments, EBOV-GP_E545D_ replicated efficiently in ferrets, resulting in significantly higher levels of viral RNA in blood at 5 DPI than WT-EBOV (Fig. 6c).

10.1128/mBio.01438-20.7 FIG S7 Clinical chemistry and hematology parameters in ferrets. Blood/plasma samples obtained at various time points from ferrets infected with 1,000 TCID_50_ of either EBOV-GP_WT_ or EBOV-GP_E545D_ were analyzed for the indicated clinical chemistry (a) or hematology (b) parameters. Data are presented for individual animals. ALT, alanine aminotransferase; ALP, alkaline phosphatase; TBIL, total bilirubin; AMY, amylase; GLOB, globulin; ALB, albumin; BUN, blood urea nitrogen; CRE, creatinine; GLU, glucose; TP, total protein; Na^+^, sodium; K^+^, potassium; Phos, inorganic phosphate; Ca^2+^, calcium; WBC, white blood cells; NEU, neutrophils; LYM, lymphocytes; MON, monocytes; PLT, platelets; RBC, red blood cells. The dotted line indicates the upper or lower detection limit for the assay. Download FIG S7, TIF file, 1.2 MB. © Crown copyright 2021. 2021 Crown This content is distributed under the terms of the Creative Commons Attribution 4.0 International license.

## DISCUSSION

Prior to 2013, few reports existed in the literature describing EBOV persistence in humans and no reports existed describing EVD recrudescence. Indeed, the relatively small number of documented EVD cases at the time, and the even smaller number of survivors, meant that EBOV persistence was rarely studied and poorly understood. Following the unprecedented 2013–2016 West African EVD outbreak, however, the number of EVD survivors rose to nearly 20,000, and with the dramatic increase in survivors, so too rose reports of EBOV persistence and atypical EVD presentations, including recrudescence (5–12, 20, 37). EBOV persistence associated with recrudescence is now appreciated as a rare but significant feature of EVD (14), although the factors that influence these processes remain to be uncovered.

Aside from human cases, there have been a handful of previously published reports describing atypical EVD in NHPs (38–40). For the most part, acute disease in these NHPs was described as relatively mild, and viremia was low. Most of the animals appeared to recover from acute disease in about 2 weeks, followed by a “relapse” and death between 19 and 28 DPI, whereas a minority of animals most likely experienced a prolonged disease from which they never truly recovered, leading to death by 14 and 16 DPI (39). Multiorgan distribution of virus at the time of death was reported for most of the animals, although viremia at terminal time points was not consistently published. In all cases, evidence of neuropathology and/or virus in the central nervous system was reported. One interpretation of these data is that the therapeutics, including antibodies, used to treat these animals may have facilitated clearance of the virus from circulation—thereby permitting initial recovery—but not from immune-privileged sites, from which virus reemerged to cause disease (39, 40).

In the case of NHP B5, the precise mechanism underlying atypical EVD, as well as its relationship to this animal’s demise, are unclear, and it is likely that multiple different factors contributed. Among these factors, the antibody treatment that the animal received may have been the most critical. E545D, which is proximal to key residues of the CA45 epitope (Fig. 4c) (30), most likely arose in response to selective pressure from antibody treatment, allowing the virus to resist neutralization and perhaps establish persistence in the first place. In this context, it is difficult to rationalize why the mutation was not detected earlier than 21 DPI—14 days after the last antibody treatment—although we suspect that it may have arisen much earlier, either at very low frequencies and/or in specific tissue compartments that were not sampled until the animal’s demise. Mutations T544I and D552N also occurred near residues of the CA45 antibody epitope; however, unlike E545D, which arose *de novo* during infection, both T544I and D552N were present in the inoculum virus and therefore unlikely to have played a role in antibody evasion, given that treatment was effective in all other animals. Indeed, the D552N mutation did not significantly affect CA45 binding.

In addition to evading mAb neutralization, the E545D mutation may have produced a slightly more virulent virus. We hypothesize that the growth advantages conferred by this mutation may be linked to the virus’s ability to cause more severe disease and that this increase in virulence contributed to disease in NHP B5. Interestingly, the E545D mutation was identified in virus obtained from a single patient during the 2013–2016 West African EBOV outbreak; however, the patient’s outcome or treatment history is unknown (41). T544I, which is adjacent to E545, has also been shown to increase both virus entry and growth kinetics in NHP cells, but it appears to be a tissue culture-specific mutation (42, 43). Whether this mutation also contributed to disease in NHP B5 remains to be investigated. Similarly, D552N, which may be linked to tissue culture adaptation, also appears to enhance *in vitro* growth kinetics (44). Whether this mutation contributed to disease in NHP B5 is unknown but perhaps unlikely considering it was present at a high frequency in the inoculum virus. Further research is required to address these issues, particularly whether any of these mutations definitively cause severe or atypical disease in NHPs and whether different combinations of these mutations (including the c7342t silent mutation) have any bearing on disease outcome. Mutations in other genes or noncoding regions mostly occurred at very low frequency, except for c440t in the NP gene promoter region that appeared at high frequency in the mesenteric lymph node and stomach samples. A partial role for these mutations in the atypical EVD reported here cannot be ruled out.

Although it remains unclear why different tissues contained virus with different sets of mutations and/or different frequencies of the same mutations, we hypothesize that these viral quasispecies were the result of adaptation to distinct selective pressures within individual tissue compartments. The nature of these selective pressures is unclear and likely related to intrinsic features of each tissue compartment as well as the half-life of the therapeutic mAbs and their ability to penetrate different tissues. Virus from the mesenteric lymph node and stomach appeared to be the most similar to each other and among the most divergent from the rest of the samples, perhaps owing to the abundance of immune cells associated with these tissues. Intriguingly, the c7342t silent mutation, along with T544I and E545D, reached frequencies of nearly 100% in virus obtained from these tissues, while D552N, as well as the silent mutation in VP30, t9267c, were undetectable, despite the relatively high frequency of both mutations in the inoculum virus. Conversely, D552N predominated in virus from the kidney, where it was the only GP mutation detected. Virus from the brain lost the T544I mutation (compared to the inoculum virus), whereas virus from the testes appeared not to have acquired E545D, instead displaying a frequency of GP mutations that closely resembled what was observed in the inoculum virus. Virus from the liver, spleen, and lung possessed all four mutations, although the frequencies of each differed depending on the tissue. Interestingly, in instances where the frequency of D552N was high, the frequency of the T544I and E545D mutations was low or undetectable, and vice versa. This relationship was particularly striking when comparing virus from the kidney to that from the mesenteric lymph node and stomach, but it was also evident in virus from most other tissues analyzed. Indeed, preliminary analyses indicated that the nucleotide mutation (g7691a) responsible for D552N rarely occurred on the same read as the nucleotide mutations (c7668t and g7672t/c) responsible for T544I and E545D, suggesting that the D552N mutation may not have existed within the same virus genome as the other two mutations and may have had an antagonistic relationship with them.

Aside from external factors—such as antibody treatment and virus genotype—that may have influenced the clinical outcome of NHP B5, the health status and genetic background of the animal itself may also have predisposed it to more severe acute disease and/or atypical progression. For instance, neutrophilic leukocytosis was evident in NHP B5 prior to infection (on day −1), with both the WBC and neutrophil counts more than twofold greater than the average count for the survivors and well outside the normal range for rhesus macaques. While neutrophilia can have many causes—including bacterial infections, certain cancers, and other stressors (45)—no evidence of these factors was found in NHP B5 prior to or during the study. It is not clear how neutrophilia *per se* could influence the severity or progression of EVD, but it could have been indicative of some underlying immune dysfunction or predisposition to a strong inflammatory response that increased the animal’s susceptibility to infection. Indeed, it is worth noting that NHP B5 exhibited pretreatment peak viral RNA levels in blood that were more than 2 log units higher than those observed in most of the other animals. Whether this high degree of virus replication was a result of immune dysfunction, host genetics, or some other factor is unknown, but it seems likely that it may have set the stage for the events that followed.

Overall, based on the pattern of viral mutations found in NHP B5, as well as its clinical background and treatment history, we envision a set of conditions that could have led to atypical EVD in this animal. Exceptionally high levels of virus replication early during infection—perhaps facilitated in part by an ineffective immune response—likely led to the dissemination of virus into a number of peripheral organs and tissues, including immune-privileged sites such as the brain and CSF, where the virus established persistence. The high level of virus replication may have also helped increase the probability of an escape mutant arising in response to various selection pressures, including the treatment antibodies. While the monoclonal antibody cocktail was sufficient to clear virus from blood and permit initial recovery, it was unlikely to have been sufficient to neutralize virus in all tissues and organs throughout NHP B5. This is supported by estimated antibody biodistribution coefficients that suggest that less than 15% of the plasma antibody concentration is deposited within tissues, with ∼12 to 14% deposited into the liver, kidneys, and spleen, ∼5 to 8% deposited into the stomach, testes, and lymph nodes, and less than 0.5% deposited in the brain (46). Thus, efficient clearance of virus from the circulation but inefficient virus clearance from tissues may have provided several sites where EBOV could continue to replicate and cause damage. Different selective pressures within these distinct organ/tissue microenvironments likely facilitated the development of viral quasispecies, some of which may have also contributed to disease. Finally, treatment with antibody CA45 in particular may have played a critical role in selecting for an antibody escape mutation (E545D) that not only allowed the virus to evade neutralization but may have also coincidentally contributed to an increase in virulence. The degree to which any one of these conditions may have influenced the outcome of NHP B5 is unclear, but it is likely that the cumulative result of a number of variables—ranging from host genetic background to virus adaptation—may have adventitiously produced the atypical EVD course reported herein.

Despite our thorough characterization of this particular instance of atypical EVD, our study has some limitations, primarily derived from the fact that the clinical outcome of NHP B5 was unexpected and is impossible to replicate. The majority of our analysis was performed well after the termination of the study, and for this reason, we do not have access to samples that, in hindsight, would be expected to shed additional light on the atypical EVD described herein. For instance, because we did not collect tissue samples from any of the surviving animals, we are unable to compare histopathology or viral load between these animals and NHP B5. Thus, we do not know whether the surviving animals displayed similar histopathologic lesions or whether they also possessed subpopulations of organ-specific viral quasispecies. Similarly, we did not assess serum from NHP B5 for its neutralizing activity against EBOV containing the GP E545D mutation. Thus, although we conclusively demonstrated that E545D escapes neutralization by mAb CA45, we cannot definitively conclude that this mutation escapes neutralization from the antibodies produced by NHP B5.

In addition to these limitations, a number of questions also remain unanswered. The true nature of the atypical disease described here remains unclear. Is this an example of virus persistence and disease recrudescence, as has been observed in some human cases? Or did NHP B5 experience a protracted disease course that culminated in death later postinfection? While the case of NHP B5 shares some similarities with reports of recrudescence in humans (11), including high initial viremia followed by clearance and eventual relapse, the duration of disease for NHP B5 was much shorter, and it remains possible that the animal never completely recovered from the initial disease. The timing of demise, potential neurologic signs (shaking, unresponsiveness), and consistent histopathologic lesions in the brain may suggest that the animal succumbed to EBOV-associated meningoencephalitis, a recognized complication of EVD seen in the second or third week of illness in humans (4). This is reminiscent of similar reports in NHPs (39, 40), as well as some human case reports (11, 37, 47, 48), and it is consistent with the extended period of weight loss observed in NHP B5. Nevertheless, we lack definitive evidence of a profound neurologic manifestation linked to EVD in this case. It is also unusual that no lesions were observed in the liver, kidney, and spleen of NHP B5, despite high levels of viral RNA in these tissues, as well as disturbances in some clinical chemistry parameters suggestive of damage to these organs. This may suggest that dysfunction in these organs at the time of euthanasia was largely mild and/or subcellular or that the lesions were absent in the tissue sections we examined. While most evidence points toward EBOV as the most likely etiological agent of this animal’s demise, it cannot be completely ruled out that NHP B5 succumbed to an unrelated illness or another complicating condition.

Regardless of the disease experienced by NHP B5, several more questions about the establishment of EBOV persistence and the development of atypical EVD are still left unanswered. For example, what specific combination of host and viral factors drive the development of persistence and, occasionally, recrudescence? At what frequency does persistence develop? What is the duration of virus persistence? And what is the probability of recrudescence or related presentations of atypical disease? Part of the difficulty in answering these questions lies in the rarity of persistence, particularly as it is observed in NHPs. A retrospective evaluation of archived tissue samples taken from rhesus macaques that had survived EBOV infection revealed that only ∼10% of the animals had evidence of virus persistence and replication in immune-privileged sites (eyes, testes, and brain) up to 43 DPI (49). Indeed, the difficulties associated with characterizing such rare events are compounded by the absence of any animal model that reliably recapitulates them. Future work should focus on the continued search for suitable model systems that accurately reproduce less well-studied facets of disease caused by EBOV, including persistence and recrudescence. Notably, our findings suggest that longer postchallenge observation of animals in NHP studies, beyond the conventional 28-day period, may be warranted. Such work will not only inform our understanding of EVD, but it will also aid in the development of EBOV countermeasures that prevent the establishment of EBOV persistence in the first place.

## MATERIALS AND METHODS

### Biosafety and animal ethics statement.

All work with infectious Ebola virus (EBOV) was performed in the containment level 4 (CL4) laboratories at the Canadian Science Centre for Human and Animal Health (CSCHAH), Public Health Agency of Canada, Winnipeg, Manitoba, in accordance with standard operating protocols. Animal experiments were reviewed and approved by the Animal Care Committee of the CSCHAH in accordance with guidelines from the Canadian Council on Animal Care. All staff working on animal experiments completed education and training programs according to the standard protocols appropriate for this biosafety level.

### Viruses and cells.

Vero E6, A549, Huh7, and Tb1.Lu cells were obtained from the American Type Culture Collection, and Huh7 cells were obtained from the Japanese Collection of Research Bioresources. All cells were maintained in Dulbecco’s modified Eagle medium (DMEM; HyClone) supplemented with 5% heat-inactivated fetal bovine serum (HI-FBS; HyClone), 2 mM l-glutamine (HyClone), as well as 50 U/ml penicillin and 50 μg/ml streptomycin (HyClone) at 37°C and 5% CO_2_. Wild-type Ebola virus variant Makona-C07 (WT-EBOV; Ebola virus H.Sapiens-wt/GIN/2014/Makona-Gueckedou-C07; GenBank accession no. KJ660347.2) was propagated once on Vero E6 cells and stored in liquid nitrogen. Recombinant EBOV (variant Makona-C07) expressing enhanced green fluorescent protein (EGFP) (WT-EBOV-EGFP; Ebola virus H.Sapiens-rec/GIN/2014/Makona-Guekedou-C07-EGFP), recombinant EBOV (variant Makona-C07) possessing mutation E545D in GP (EBOV-GP_E545D_; Ebola virus H.Sapiens-rec/GIN/2014/Makona-Gueckedou-C07-GP_E545D_), and recombinant EBOV (variant Makona-C07) expressing EGFP and possessing mutation E545D in GP (EBOV-EGFP-GPE545D; Ebola virus H.Sapiens-rec/GIN/2014/Makona-Gueckedou-C07-EGFP-GP_E545D_) were generated using standard reverse genetics procedures as described previously (50), propagated on Vero E6 cells, and stored in liquid nitrogen. All virus sequences were confirmed by next-generation sequencing as described below.

### Nonhuman primate study and design.

Seventeen rhesus macaque nonhuman primates (NHPs) of Chinese origin were randomly assigned into one of four groups based on gender and weight (see Table S1 in the supplemental material). This study was not performed in a blind manner. Prior to infection, animals were housed in an animal biosafety level 2 (ABSL-2) room, according to approved protocols, with temperature and humidity monitored and maintained at 69 to 70°F and 24 to 59%, respectively. Automated light controls were used for light/dark cycles of approximately 12 h. Acclimation to the ABSL-2 environment lasted for at least 7 days before the animals were transferred to CL4 and inoculated with virus. Prior to and during the study, animals were provided water *ad libitum*, and they were fed commercially available monkey chow at least daily. Fruits, vegetables, and other treats were also provided. Environmental enrichment was provided in the form of visual stimulation and toys. Procedures involving manipulation, including phlebotomy or physicals, were performed while the animal was under anesthesia.

All NHPs were inoculated with a dose of 1,000 TCID_50_ (back titered to 734 TCID_50_) WT-EBOV via the intramuscular (IM) route (Table S1). Animals in group A (*n* = 5) and group B (*n* = 5) were treated with a single dose of a monoclonal antibody cocktail containing 20 mg each of antibodies FVM04 and CA45/kg of body weight at 4 days postinfection (DPI); group B animals also received a second treatment, delivered on 7 DPI, of the same antibody cocktail containing 10 mg/kg of each antibody. Animals in group C (*n* = 5) were treated with a single dose of a monoclonal antibody cocktail containing 20 mg/kg each of antibodies FVM04 and CA45, in addition to 50 mg/kg of antibody MR191 on 4 DPI, and they received a second treatment on 7 DPI with a single dose of antibody cocktail containing 10 mg/kg FVM04 and CA45, as well as 50 mg/kg MR191. Animals in the control group (*n* = 2) received equivalent volumes of vehicle control (20 mM citrate, 10 mM glycine, 8% sucrose, 0.01% polysorbate-80 at pH 5.5) on 4 and 7 DPI. Administration of antibody cocktails or vehicle control was delivered by intravenous (IV) bolus via slow syringe push lasting 3 to 5 min into the saphenous vein. Following inoculation, NHPs were observed at least twice daily until onset of symptoms, after which they were observed at least thrice daily. Observations were documented using an NHP Humane Endpoint Clinical Scoring Chart approved by the Animal Care Committee and included responsiveness and recumbence, food consumption, condition of stool, presence of urine output, respiratory difficulty, as well as the presence of rash, bleeding, cyanosis, hemorrhage, or exudates. On predetermined days (0, 4, 7, 10, 15, 21, and 28 DPI), animals were anesthetized and given a physical examination by qualified study personnel. Weight and body temperature measurements were also taken at these time points, as were blood and swab (oral, nasal, rectal) samples. Animals that reached the humane clinical endpoint score were euthanized, and terminal blood samples were collected. In the case of NHP B5, which was euthanized on 26 DPI, a necropsy was performed, and tissue samples were collected. The disease and treatment course for the control animals, as well as those in groups A and C, have been described, in detail, elsewhere (29).

### Ferret study and design.

Fourteen 6-month-old male and female domestic ferrets (Mustela putorius furo) were purchased from Marshall BioResources (New York, USA) and had been treated with anticoccidials as well as vaccinations against distemper and rabies. Each ferret was also implanted with an IPTT-300 temperature and identification (ID) transponder (BioMedic Data Systems Inc., USA) subcutaneously over the dorsal aspect of the caudal region. Per protocol, animals were acclimated for at least 7 days before being transferred to CL4 and inoculated with virus. Prior to and during the study, animals were given food and water *ad libitum* and monitored at least twice daily. Environmental enrichment was provided in the cages for the duration of the experiment.

At the commencement of the experiments, equal numbers of male and female ferrets were randomly assigned into one of two groups and inoculated intramuscularly with 1,000 TCID_50_ of WT-EBOV (*n* = 4) or EBOV-GP_E545D_ (*n* = 6). Following inoculation, animals were monitored daily for signs of disease that included changes in body weight, temperature (via transponder reading), physical activity, and food and water intake. Observations were documented on a Ferret Humane Endpoint Clinical Scoring Chart approved by the Animal Care Committee. On predetermined days (−2, 3, 5, and 7 DPI) or when an animal reached a clinical score indicating euthanasia, animals were anesthetized and given a physical examination by qualified study personnel. Weight and body temperature measurements were also taken at these time points, as were blood and swab (oral, nasal, rectal) samples. Animals that reached the humane clinical endpoint score were euthanized, and terminal blood samples were collected. Historical control animals (*n* = 4), which were inoculated with WT-EBOV and manipulated exactly as described above, were also included in our data set to increase statistical confidence.

### EBOV RNA quantification.

Viral RNA was extracted from blood and swab samples using the QIAmp Viral RNA minikit (Qiagen) and from tissues using the RNeasy minikit (Qiagen), essentially according to the manufacturer’s directions. Viral RNA was assessed using a reverse transcription-quantitative PCR (RT-qPCR) assay, as described previously (50, 51). All RT-qPCRs were performed on the LightCycler 480 thermal cycler using the LightCycler 480 RNA Master Hydrolysis Probes kit (Roche) and the following primers and probe: forward primer, CAGCC AGCAA TTTCT TCCAT; reverse primer, TTTCG GTTGC TGTTT CTGTG; probe, 6-carboxyfluorescein (FAM)-ATCAT TGGCG TACTG GAGGA GCAG-BHQ1 (black hole quencher 1). Cycling conditions were as follows: 63°C for 3 min and 95°C for 30 s, followed by 45 cycles with 1 cycle consisting of 95°C for 15 s and 60°C for 30 s. Virus genome equivalents (GEQ) per milliliter were calculated from a standard curve.

### Infectious virus quantification.

Vero E6 cells were seeded in 96-well plates (Corning) for 95% confluence at the time of infection. Tissue homogenates were serially diluted 10-fold in DMEM containing 2% HI-FBS, and 100 μl of each dilution was used to inoculate cells, in triplicate. Virus was allowed to attach for 1 h at 37°C before the supernatant was removed and replaced with 100 μl DMEM supplemented with 2% HI-FBS, 1% penicillin/streptomycin, and 2 mM l-glutamine. Cells were incubated for 14 days, during which time they were monitored for cytopathic effect (CPE) and 50% tissue culture infectious dose (TCID_50_) was calculated for each sample using the Reed and Muench method.

### Histopathology, immunohistochemistry, and *in situ* hybridization.

Tissue samples were collected and fixed in 10% neutral buffered formalin, embedded in paraffin, sectioned, and stained with hematoxylin and eosin. For immunohistochemistry, paraffin-embedded tissue sections were quenched for 10 min in aqueous 3% H_2_O_2_ and then pretreated with proteinase K for 10 min. The primary antibody was rabbit polyclonal anti-EBOV VP40 that was used at a 1:2,000 dilution for 1 h. The tissue sections were visualized using a horseradish peroxidase-labeled polymer Envision + system (anti-rabbit; Dako) and reacted with the chromogen diaminobenzidine. The sections were counterstained with Gill hematoxylin. For *in situ* hybridization, 5-μm paraffin-embedded tissue sections were cut, air dried, and melted onto charged slides in a 60°C oven. The slides were cleared in xylene and 100% ethanol and then air dried. The sections were quenched for 10 min in aqueous H_2_O_2_, boiled in target retrieval solution for 15 min, rinsed in 100% ethanol, and air dried. A final pretreatment of protease plus enzyme was applied for 30 min at 40°C. The probe (Makona VP-40, from Advanced Cell Diagnostics) was applied and incubated at 40°C for 2 h. Hybridization amplification steps (AMP 1-6) were applied to the slides for the recommended times and temperatures as per the manual for the RNAscope 2.5HD detection reagent – brown kit (ACD). Signal was visualized by the chromogen diaminobenzidine (DAB). The sections were then counter stained with Gill’s hematoxylin, dehydrated, cleared, and coverslipped.

### Blood biochemistry and hematology.

NHP and ferret blood biochemistry was evaluated using the VetScan VS2 chemistry analyzer (Abaxis) using serum separated from heparinized blood via centrifugation. Concentrations of the following analytes were measured: alanine aminotransferase (ALT), alkaline phosphatase (ALP), total bilirubin (TBIL), amylase (AMY), globulin (GLOB), albumin (ALB), blood urea nitrogen (BUN), creatinine (CRE), glucose (GLU), total protein (TP), sodium (Na^+^), potassium (K^+^), inorganic phosphate (Phos), and calcium (Ca^2+^). Complete NHP and ferret blood cell counts were determined using the VetScan HM5 hematology analyzer (Abaxis) from EDTA whole blood. The following cells and components were quantified: white blood cells (WBC), neutrophils (NEU), lymphocytes (LYM), monocytes (MON), platelets (PLT), red blood cells (RBC). All analyses were performed according to the manufacturer’s directions.

For the NHP data, normal ranges for each analyte are presented as gray boxes. The average mean values of each analyte for uninfected, healthy, adult rhesus macaques were calculated from historical data taken from previous experiments performed at the Public Health Agency of Canada in addition to published reports of macaque blood biochemistry and hematology values (52, 53). The normal ranges encompass the average mean value for a given analyte plus and minus the pooled standard deviations and were derived from 59 to 152 (mean = 103, median = 95) different male and female rhesus macaques, depending on the analyte.

### IgG and IgM ELISAs.

IgG and IgM levels in NHP serum were measured by enzyme-linked immunosorbent assay (ELISA) as described elsewhere (54). Briefly, 96-well plates (Corning) were coated with recombinant EBOV GPΔTM (IBT Bioservices), and blocked with phosphate-buffered saline (PBS) containing 5% skim milk. NHP serum samples and the in-house standards were diluted in PBS containing 2% skim milk before being applied to the plate, incubated, and then washed three times in PBS plus 0.1% Tween 20 using a BioTek plate washer. Goat anti-monkey IgG or goat anti-monkey IgM secondary antibodies conjugated to horseradish peroxidase were used at a concentration of 1 μg/ml, and following a second set of washes, the plate was incubated with 3,3′,5,5′-tetramethylbenzidine (TMB) Single Solution substrate (Thermo Fisher Scientific). Absorbance was read at 650 nm using a BioTek Synergy HT plate reader (BioTek), and samples for which all dilutions fell outside the limits of detection were reevaluated at higher or lower dilutions, as appropriate. Data were fit to the standard curve and are displayed as arbitrary antibody units (AU) per milliliter. Note that the therapeutic antibodies CA45 and FVM04 possess human Fc domains and therefore do not cross-react with the secondary antibodies used in this assay (30, 31).

### Cytokine analyses.

The NHP cytokine response was quantified in γ-irradiated (5 mrad) serum samples using the Cytokine 29-Plex Monkey Panel (Thermo Fisher Scientific) and the Luminex MAGPIX instrument (Thermo Fisher Scientific) according to the manufacturer’s directions and as described elsewhere (55). Briefly, serum was diluted 1:4 and added to 1× anti-cytokine antibody-coupled beads. Following incubation, 1× biotinylated detector antibody was added, after which samples were incubated with 1× streptavidin-R phycoerythrin (RPE) solution. Fifty beads, resuspended in wash buffer, were counted for each sample. All samples were run in duplicate, and mean fluorescence intensity was used to calculate final concentrations in picograms per milliliter. The following cytokines were analyzed: gamma interferon (IFN-γ), tumor necrosis factor alpha (TNF-α), interleukin-1 receptor antagonist (IL-1RA), IL-1β, IL-2, IL-4, IL-5, IL-6, IL-8 (CXC chemokine ligand 8 [CXCL8]), IL-10, IL-12, IL-15, IL-17, monokine induced by IFN-γ (MIG; CXCL9), macrophage migration inhibitory factor (MIF), monocyte chemoattractant protein 1 (MCP-1; CC chemokine ligand 2 [CCL2]), macrophage inflammatory protein 1α (MIP-1α; CCL3), MIP-1β (CCL4), macrophage-derived chemokine (MDC; CCL22), IFN-inducible protein 10 (IP-10; CXCL10), eotaxin, regulated-on activation normal T-cell expressed and secreted (RANTES; CCL5), IFN-inducible T-cell alpha chemoattractant (ITAC), epidermal growth factor (EGF), basic fibroblast growth factor (bFGF), hepatocyte growth factor (HGF), vascular endothelial growth factor (VEGF), granulocyte colony-stimulating factor (G-CSF), and granulocyte-macrophage CSF (GM-CSF). Cytokine data from all animals except NHP B5 made up part of a combined data set published elsewhere (55).

### Next-generation sequencing.

Viral RNA was subjected to reverse transcription with the SuperVilo enzyme (Thermo Fisher) according to the manufacturer’s instructions. Subsequent PCRs were carried out using CloneAmp HiFi PCR Premix (Clontech) with the following conditions: 98°C for 30 s, 39 cycles with 1 cycle consisting of 98°C for 10 s, 65°C for 15 s, and 72°C for 1 min 30 s, and a final extension at 72°C for 10 min. Amplicons were purified by gel electrophoresis and quantified using the Qubit Fluorometric Quantification (Thermo Fisher). Library construction was done with the Nextera DNA XT sample preparation kit (24-sample) (Illumina) as per manufacturer’s instructions, followed by sequencing using a MiSeq sequencer (Illumina) with the MiSeq reagent kit v3 (600 cycles) (Illumina). Data analysis was performed as follows. Adaptor sequences were trimmed using the FASTX-Toolkit, and reads shorter than 25 nucleotides were discarded. Paired sequencing data were mapped to the EBOV (variant Makona) virus genome (GenBank accession no. KJ660347.2) using the Bowtie aligner (56), with the max mismatches parameter “-v” set to 2, the “-q” parameter to handle fastq files, and the parameter “-X” for maximum insert size for valid paired-end alignments set at 800. Resulting alignments were sorted using the SAMtools package (57). Variant calling used the LoFreq program (58) to identify single nucleotide polymorphisms (SNPs) that had low false-discovery rate values, controlling for sequencing coverage and for read qualities. Minimum read coverage per position was 10, while the minimum Phred quality score for single nucleotide variants (SNVs) was 58. The read coverage at each position was assessed using the SAMtools “depth” command and sorted for positive-sense and negative-sense alignments using the awk utility. Analysis of individual point mutation occurrences was performed using custom Python and R scripts. Read coverage plots per gene were created using custom R scripts. Mutation frequencies were calculated as the number of reads at a given nucleotide position with a specific mutation divided by the total number of reads at the same nucleotide position. Results are depicted as heatmaps, created using GraphPad Prism (version 8).

### Antibody reactivity ELISAs and bio-layer interferometry.

WT-EBOV (variant Makona) GP ectodomain (GPΔTM_WT_) or GP ectodomains harboring the E545D or D552N mutation (GPΔTM_E545D_ and GPΔTM_D552N_, respectively) were expressed in S2 cells (ExpreS2ion Biotechnologies, Denmark) and purified using a 6× histidine tag. Monoclonal antibody reactivity to these proteins was then measured using a standard ELISA. Briefly, GP was coated overnight onto Maxisorp 96-well plates (Nunc) at 1 μg/ml concentration in Dulbecco’s PBS (DPBS) at 4°C. The following day, plates were blocked with StartingBlock T20 (PBS) blocking buffer (Thermo Fisher Scientific) for 1 h at ambient temperature. Antibodies serially diluted in StartingBlock were then incubated onto plates for 1 h followed by incubation with a respective anti-human or anti-mouse FC antibody (KPL) for 1 h. Each of these steps was interspersed by three consecutive washes with DPBS containing 0.05% Tween using a plate washer. For readout, plates were incubated with TMB substrate (Life Technologies) for 30 min prior to readout at an optical density at 650 nm (OD_650_) using a VersaMax plate reader.

All bio-layer interferometry (BLI) kinetic measurements were made using an Octet Red96 instrument. Briefly, GPΔTM_WT_, GPΔTM_E545D_, or GPΔTM_D552N_ was diluted in 1× kinetic buffer (KB) (Fortebio) in flat, black 96-well plates at 25°C shaking at 1,000 rpm. Monoclonal antibody CA45 was diluted to 1 μg/ml in KB and loaded onto hydrated protein G sensors (Fortebio) for 120 s. After loading, a 60-s baseline was established to ensure stable ligand binding before the coated sensors interacted with wells serially diluted with GP analyte. The association was maintained for 300 s, followed by a 300-s dissociation step. To correct for nonspecific binding of GP to sensors in the absence of antibody, reference sensors were used in parallel for each concentration of GP and then subtracted from the total response. ForteBio Data Analysis v9 software was used for data analysis which allowed for the calculation of *k*_on_, *k*_off_, and *K_D_* values using the global fit 1:1 Langmuir binding model.

### Antibody neutralization assay.

Neutralization assays with replication-incompetent pseudotyped vesicular stomatitis virus (VSV) expressing a luciferase reporter and either WT-EBOV GP (GP_WT_) or GP containing the E545D mutation (GP_E545D_) were performed as described previously (31). Briefly, a serial dilution of antibody was incubated with pseudotyped virus in serum-free Eagle’s minimum essential medium (EMEM) for 1 h at room temperature before Vero cells were infected at a multiplicity of infection (MOI) of 0.04. One hour following infection, cells were supplemented with 50% (vol/vol) EMEM containing 2% FBS, 100 IU/ml penicillin, and 100 μg/ml streptomycin. Twenty-four hours later, cells were lysed for 30 min at room temperature with Passive Lysis Buffer (Promega) before the addition of Luciferase Activating Reagent (Promega). Luciferase luminescence was read using a Biotek Plate Reader, and percent neutralization was calculated based on wells containing virus only.

### EBOV *in vitro* growth kinetics.

To assess viral growth kinetics in different cell lines, an EGFP reporter gene assay was performed. Vero E6, A549, Huh7, or Tb1.Lu cells were seeded in a clear-bottom, black 96-well plate (Corning) to a density of ∼6.25 × 10^4^ cells per well at the time of infection. Cells were inoculated with either WT-EBOV-EGFP or EBOV-EGFP-GP_E545D_ at a MOI of 0.05 or 0.01 in a volume of 20 μl DMEM. Following incubation at 37°C for 1 h, the inoculum was removed, cells were washed with 50 μl DMEM, and the medium was replaced with 200 μl DMEM supplemented with 2% HI-FBS, 2 mM l-glutamine, 50 U/ml penicillin, and 50 μg/ml streptomycin. At 1, 2, 3, 4, 5, 6, 7, 8, and 11 DPI, EGFP fluorescence was measured using a Synergy XT microplate reader (BioTek), and data are displayed as the average relative fluorescence units (RFU) from three technical replicates over time.

### Statistical and other analyses.

All graphs/heatmaps were generated using GraphPad Prism (version 8), and all statistical analyses were performed using the same software. Statistical comparisons of RFU (Fig. 6a) and GEQ/ml (Fig. 6c) between WT and recombinant EBOV were performed using the unpaired, parametric, two-tailed *t* test. Statistical comparison of the Kaplan-Meier survival curves from the ferret pathogenesis experiment (Fig. 6b) was performed using the log rank and Gehan-Breslow-Wilcoxon tests. *P* values less than or equal to 0.05 are indicated with a single asterisk, less than or equal to 0.01 with two asterisks, less than or equal to 0.001 with three asterisks, and less than or equal to 0.0001 with four asterisks.
