# Supplementary material for: Atypical Ebola Virus Disease in a Nonhuman Primate following Monoclonal Antibody Treatment Is Associated with Glycoprotein Mutations within the Fusion Loop
Source: mBio. 2021 Jan 12;12(1):e01438-20. doi: 10.1128/mBio.01438-20 (PMC7844533; doi:10.1128/mBio.01438-20)
Supplement: TABLE S1 [file mBio.01438-20-st001.docx]

| **Supplementary Table 1: Study Outline** | | | | | | | | | |
| --- | --- | --- | --- | --- | --- | --- | --- | --- | --- |
| **Group** | **Study ID** | **Animal ID** | **Age**^1^ | **Sex** | **Weight**^2^ | **EBOV Dose (TCID_50_)** | **Treatment** | | **Outcome** |
|  |  |  |  |  |  |  | **4 DPI** | **7 DPI** |  |
| A | A1 | 1318 | 45 | F | 4.03 | 1000 | 20 mg/kg FVM04  +  20 mg/kg CA45 | - | Survived |
|  | A2 | 1088 | 45 | F | 3.75 | 1000 |  |  | Survived |
|  | A3 | 6126 | 45 | F | 4.03 | 1000 |  |  | Survived |
|  | A4 | 1121 | 57 | M | 4.22 | 1000 |  |  | Survived |
|  | A5 | 1227 | 45 | M | 4.53 | 1000 |  |  | Survived |
| B | B1 | 6132 | 46 | F | 4.10 | 1000 | 20 mg/kg FVM04  +  20 mg/kg CA45 | 10 mg/kg FVM04  +  10 mg/kg CA45 | Survived |
|  | B2 | 6166 | 45 | F | 3.89 | 1000 |  |  | Survived |
|  | B3 | 6300 | 45 | F | 3.40 | 1000 |  |  | Survived |
|  | B4 | 1333 | 46 | M | 4.00 | 1000 |  |  | Survived |
|  | B5 | 2025 | 45 | M | 4.58 | 1000 |  |  | Euthanized, 26 DPI |
| C | C1 | 6226 | 46 | F | 4.71 | 1000 | 20 mg/kg FVM04  +  20 mg/kg CA45  +  50 mg/kg MR191 | 10 mg/kg FVM04  +  10 mg/kg CA45  +  50 mg/kg MR191 | Survived |
|  | C2 | 6302 | 45 | F | 3.49 | 1000 |  |  | Survived |
|  | C3 | 6308 | 45 | F | 3.59 | 1000 |  |  | Survived |
|  | C4 | 6155 | 45 | M | 3.99 | 1000 |  |  | Survived |
|  | C5 | 6305 | 45 | M | 3.58 | 1000 |  |  | Survived |
| Control | Control 1 | 1500 | 51 | F | 4.40 | 1000 | Buffer | Buffer | Euthanized, 7 DPI |
|  | Control 2 | 6107 | 45 | M | 3.85 | 1000 |  |  | Euthanized, 8 DPI |
| 1. Approximate age in months  2. Weight in kilograms at beginning of study  DPI, days post-infection | | | | | | | | | |
