# Supplementary material for: Atypical Ebola Virus Disease in a Nonhuman Primate following Monoclonal Antibody Treatment Is Associated with Glycoprotein Mutations within the Fusion Loop
Source: mBio. 2021 Jan 12;12(1):e01438-20. doi: 10.1128/mBio.01438-20 (PMC7844533; doi:10.1128/mBio.01438-20)
Supplement: TABLE S2 [file mBio.01438-20-st002.docx]

| **Supplementary Table 2: Clinical History of NHP B5** | | |
| --- | --- | --- |
| **DPI** | **Inoculation/Treatments** | **Clinical Features** |
| 0 | Inoculated with 1000 TCID_50_ EBOV |  |
| 4 | Treated with 20 mg/kg FVM04 & 20 mg/kg CA45 | High levels of viral RNA in blood; slight increase in body temperature |
| 7 | Treated with 10 mg/kg FVM04 & 10 mg/kg CA45 | Mild anorexia |
| 8 |  | Mild anorexia; diarrhea |
| 9 |  | Diarrhea |
| 10 |  | Diarrhea; weight loss first noted |
| 11 |  | Diarrhea |
| 12 |  | Soft stool |
| 20 |  | Soft stool |
| 21 |  | Decreased activity; mild depression; mild anorexia; elevated body temperature; soft stool; viral RNA no longer detectable in blood |
| 22 |  | Decreased activity; mild depression; mild anorexia; soft stool |
| 23* |  | Decreased activity; mild depression; mild anorexia |
| 24 | Treated with 5 mg/kg enrofloxacin | Huddled/shaking in cage; moderate anorexia; mild depression |
| 25 | Treated with 5 mg/kg enrofloxacin | Huddled/shaking in cage; difficulty eating/drinking; decreased urine/feces output; moderate depression; visibly thin |
| 26 | Euthanized | Recumbent; not moving/unresponsive; no food/water intake; decreased respiration rate; visibly thin; hypothermia |
| * Animal separated from cage mate | | |
