# Supplementary figures and images for: Atypical Ebola Virus Disease in a Nonhuman Primate following Monoclonal Antibody Treatment Is Associated with Glycoprotein Mutations within the Fusion Loop
Source: mBio. 2021 Jan 12;12(1):e01438-20. doi: 10.1128/mBio.01438-20 (PMC7844533; doi:10.1128/mBio.01438-20)

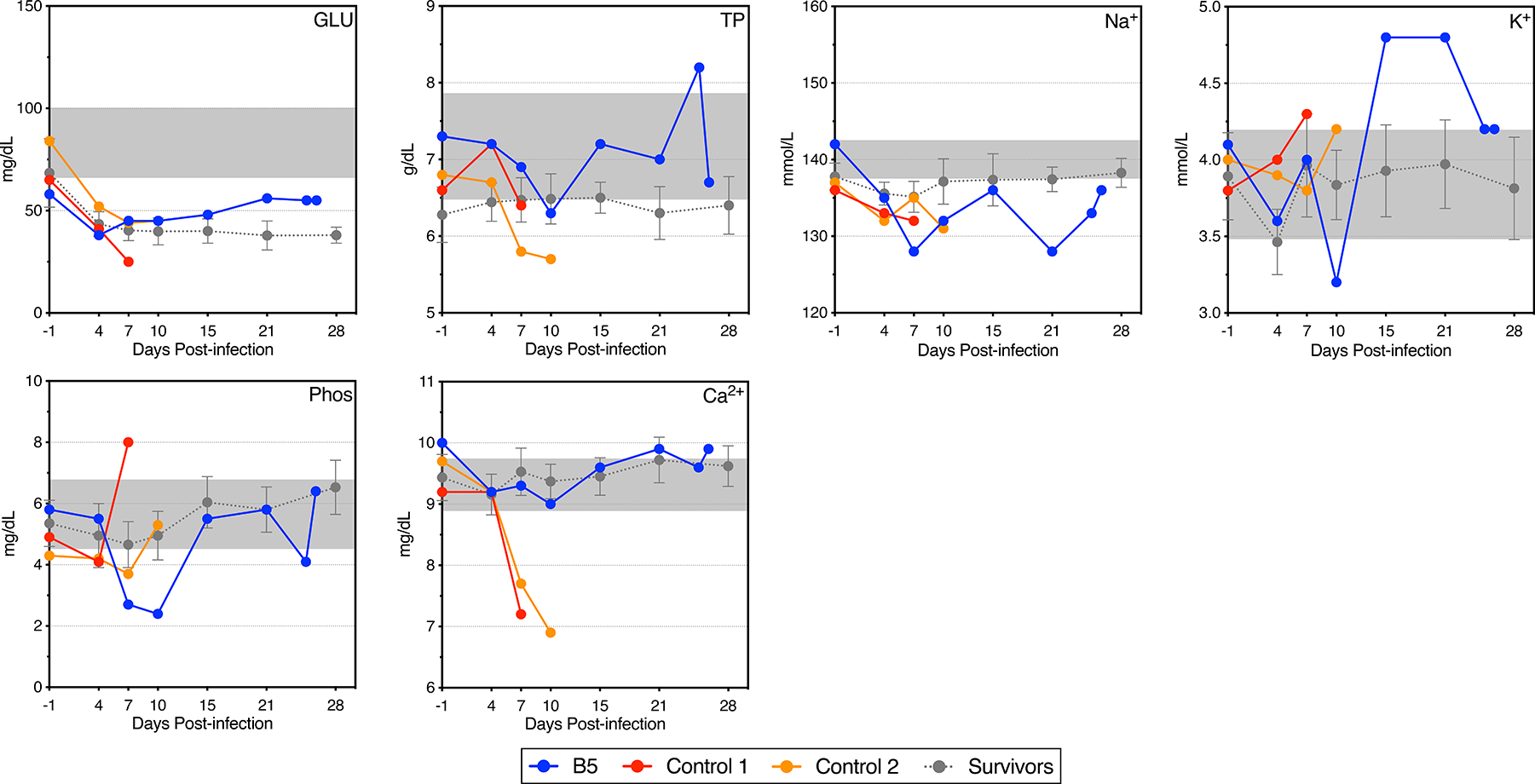

Supplement: FIG S2 [file mBio.01438-20-sf002.tif]

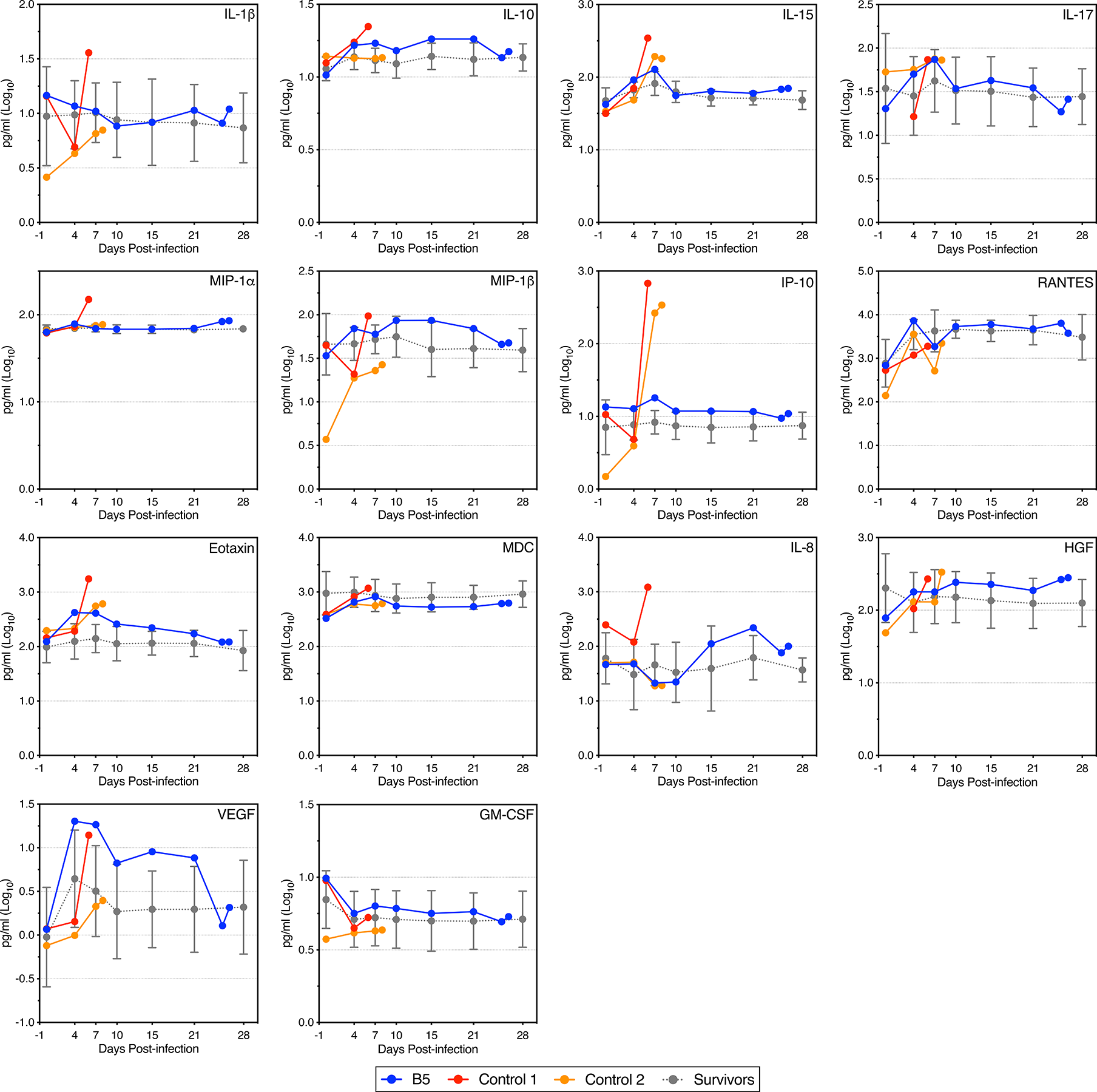

Supplement: FIG S3 [file mBio.01438-20-sf003.tif]

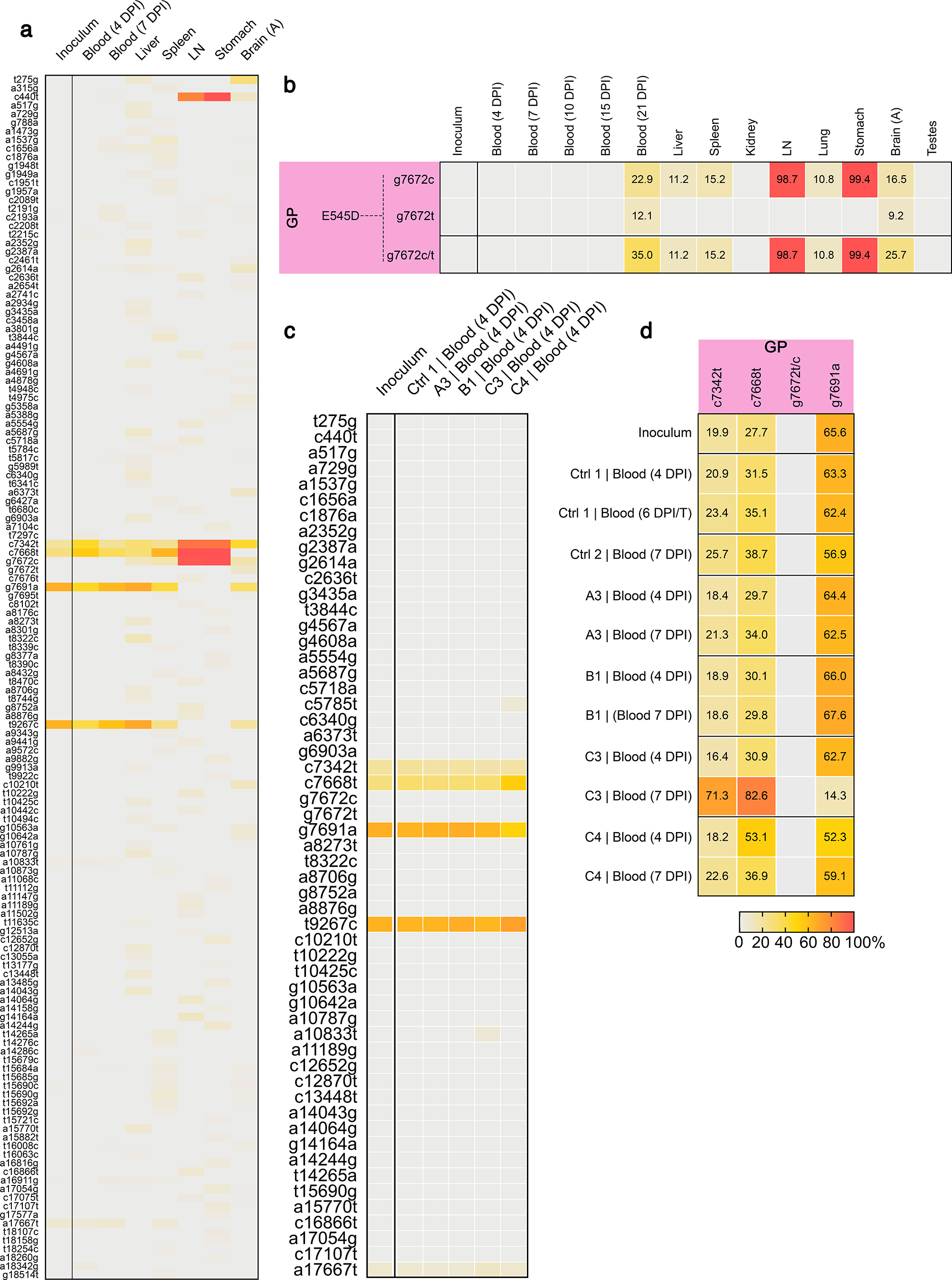

Supplement: FIG S4 [file mBio.01438-20-sf004.tif]

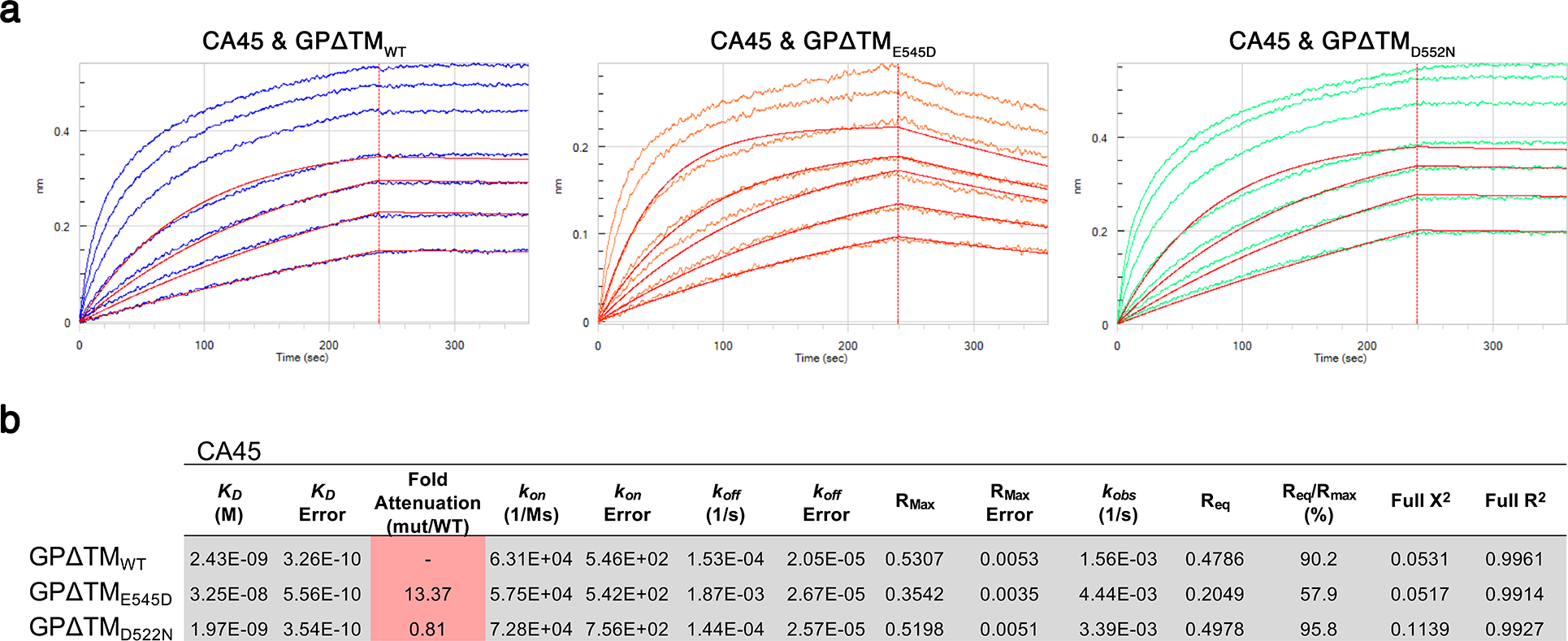

Supplement: FIG S5 [file mBio.01438-20-sf005.tif]

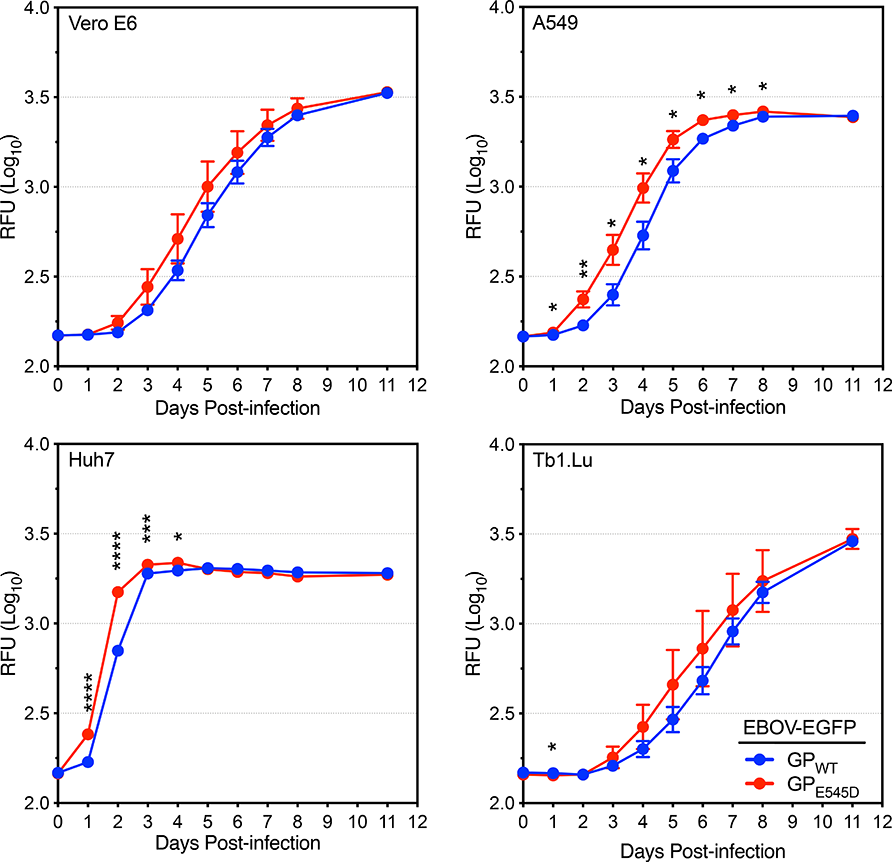

Supplement: FIG S6 [file mBio.01438-20-sf006.tif]

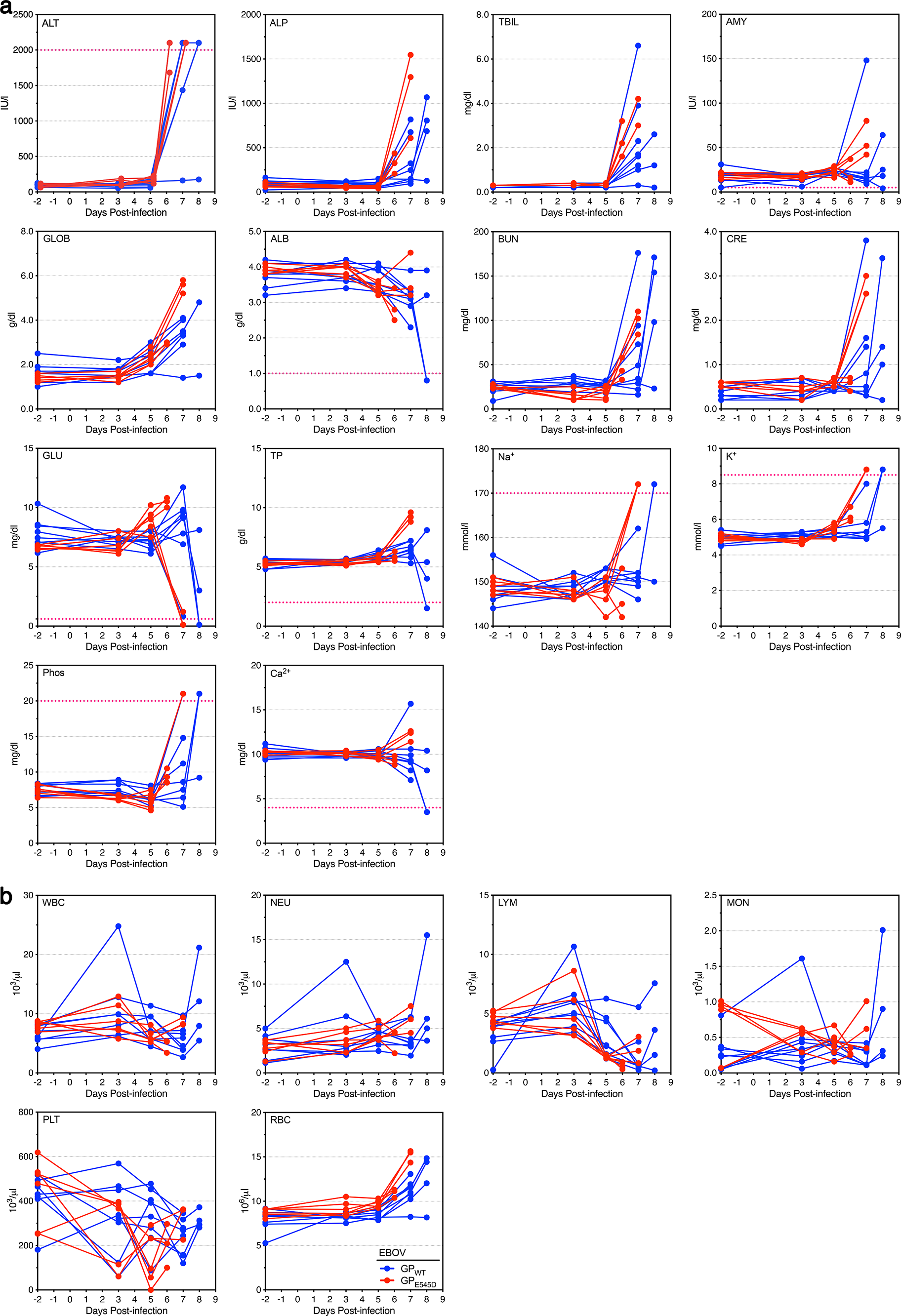

Supplement: FIG S7 [file mBio.01438-20-sf007.tif]
